# Bioinspired Nanocomposites with Self‐Adaptive Stress Dispersion for Super‐Foldable Electrodes

**DOI:** 10.1002/advs.202103714

**Published:** 2021-11-17

**Authors:** Guangtao Zan, Tong Wu, Zhenlei Zhang, Jing Li, Junchen Zhou, Feng Zhu, Hanxing Chen, Ming Wen, Xiuchun Yang, Xiaojun Peng, Jun Chen, Qingsheng Wu

**Affiliations:** ^1^ School of Chemical Science and Engineering Institute of Advanced Study Shanghai Key Laboratory of Chemical Assessment and Sustainability School of Materials Science and Engineering School of Aerospace Engineering and Applied Mechanics Tongji University Shanghai 200092 P. R. China; ^2^ School of Chemistry and Chemical Engineering Chongqing University Chongqing 400044 P. R. China; ^3^ State Key Laboratory of Fine Chemicals Dalian University of Technology Dalian 116024 P. R. China; ^4^ Department of Bioengineering University of California Los Angeles Los Angeles CA 90095 USA

**Keywords:** biomimetic, C/FeOOH, free‐standing electrodes, self‐adaptive stress dispersion, super‐foldable electronics

## Abstract

In flexible electronics, appropriate inlaid structures for stress dispersion to avoid excessive deformation that can break chemical bonds are lacking, which greatly hinders the fabrication of super‐foldable composite materials capable of sustaining numerous times of true‐folding. Here, mimicking the microstructures of both cuit cocoon possessing super‐flexible property and *Mimosa* leaf featuring reversible scatheless folding, super‐foldable C‐web/FeOOH‐nanocone (SFCFe) conductive nanocomposites are prepared, which display cone‐arrays on fiber structures similar to *Mimosa* leaf, as well as non‐crosslinked junctions, slidable nanofibers, separable layers, and compressible network like cuit cocoon. Remarkably, the SFCFe can undergo over 100 000 times of repeated true‐folding without structural damage or electrical conductivity degradation. The mechanism underlying this super‐foldable performance is further investigated by real‐time scanning electron microscopy folding characterization and finite‐element simulations. The results indicate its self‐adaptive stress‐dispersion mechanism originating from multilevel biomimetic structures. Notably, the SFCFe demonstrates its prospect as a super‐foldable anode electrode for aqueous batteries, which shows not only high capacities and satisfactory cycling stability, but also completely coincident cyclic voltammetry and galvanostatic charge–discharge curves throughout the 100 000 times of true‐folding. This work reports a mechanical design considering the self‐adaptive stress dispersion mechanism, which can realize a scatheless super‐foldable electrode for soft‐matter electronics.

## Graphical abstract

Bioinspired C‐web/FeOOH‐nanocone nanocomposites are for the first time prepared, and achieve a scatheless super‐foldable property. They can bear over 100 000 times repeated true‐folding without structure fracture, conductivity degradation, or electrochemical property change. Such breakthrough performance results from their self‐adaptive stress dispersion mechanism of biomimetic structures, which is unveiled through real‐time scanning electron microscopy (SEM) folding observation and corresponding mechanical simulations.

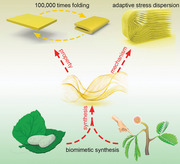

## 1 Introduction

**Figure 1:**
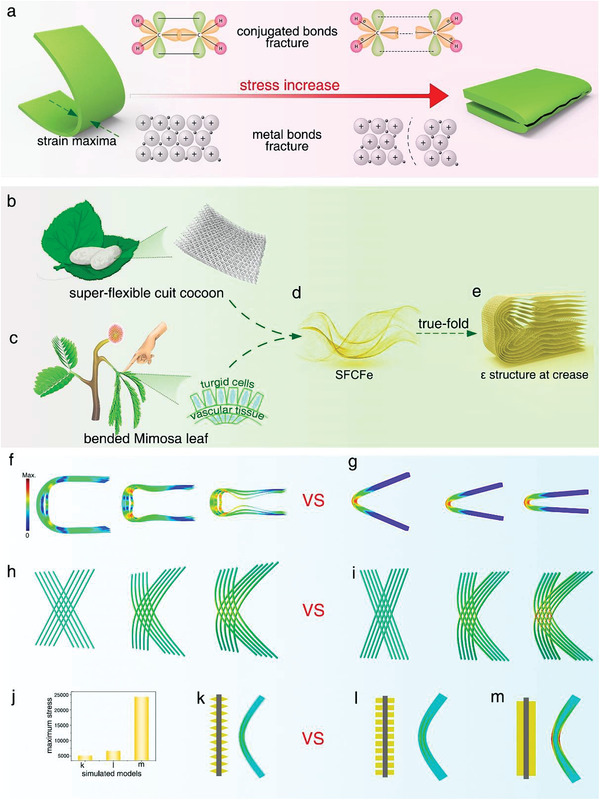
Super‐foldable SFCFe and its design principle. a) Schematic of short‐range force limit of chemical bonds in conventional conductive materials incapable of bearing repeated true‐folding. b–e) Biomimetic structural design of SFCFe enabling numerous true‐folding. f–m) Mechanical simulations showing the relationship between structure and stress at different hierarchies: (f, g) stress distributions in different 3D folding structures at crease, (h, i) stress distributions in bent 2D layers of different link types, (j–m) maximum stress values and stress distributions in bent nanofibers of different composite structures.

Foldable electronics has drawn increasing attention from concept to market.^[^ ^1^, ^2^, ^3^, ^4^, ^5^, ^6^ ^]^ It has become evident that the conventional bendable pattern cannot satisfy the high requirements in the practical application of flexible electronics owing to reliability and safety issues at unintentional extreme deformation of flexible electronic products. The increasingly functional requirements, such as wearability, implantability, and portability, of flexible products also promote the eager for super‐foldable electronic materials capable of sustaining multiple true‐folding tests (Figure S1, Supporting Information).^[^ ^7^, ^8^, ^9^ ^]^ Flexible electronic materials are widely known to be the key components of flexible devices. Typically, they present the structures of functional matters inlaying on flexible conductive bases, which have been extensively studied for flexible electrodes, sensors, triboelectric nanogenerators, etc.^[^ ^10^, ^11^, ^12^, ^13^, ^14^, ^15^, ^16^ ^]^ To improve the flexibility to foldability, great effort has been made from the structural design of both flexible conductive bases and composite structures. Unfortunately, the super‐foldable performance are so challenging that it has not been attained thus far. This is because the local stress generated at the crease under true‐folding extremely exceeds that under bending or pseudo‐folding by several orders of magnitude (Figure S1, Supporting Information). Considerable stress definitely causes damage to chemical bonds, eventually leading to fracture during the repeated folding of conductive bases without special structural design. This is attributed to the short‐range‐force nature and incapability of chemical bonds to sustain large deformations (**Figure** **1**a; Note S1, Supporting Information).^[^ ^17^, ^18^ ^]^ In addition, the realization of high functional performance while maintaining the super‐foldability of electronic materials must also be considered. In view of these, at least four problems must be solved in their design. (1) Super‐foldable conductive bases must be prepared. (2) How can a firm bond that prevents detachment between functional materials and super‐foldable bases be achieved? (3) How can the super‐foldability of bases be protected from the negative effect of inlaying functional materials? (4) The electrochemical/electronic performance based on the foregoing must be maximized. Consequently, the attainment of super‐foldable and high‐performance electronic materials remains extremely problematic.

To improve the flexibility of electronic materials, different hierarchical structures have been designed. Pore engineering is a widely adopted strategy for improving flexibility. For example, macropores have been introduced into carbon web nanofibers, and the resultant bamboo‐like and golden‐toad‐egg‐like nanofiber webs have been demonstrated to have high flexibility and even foldability over a limited times.^[^ ^19^, ^20^ ^]^ The presence of numerous micropores and mesopores has also been proved to enable materials to sustain multiple folding.^[^ ^21^ ^]^ The adjustment strategy for interlaminar interactions is also found effective in boosting flexibility. Some studies have indicated that the increase in interlaminar interactions can result in considerable tensile strength, and provide materials with good mechanical flexibility. These materials include cross‐linked N‐doped carbon nanofiber/Polyaniline networks, graphene films with sequential ionic and *π* bridging, and MXene/graphene films.^[^ ^22^, ^23^, ^24^ ^]^ While other studies have demonstrated the effectiveness of using separable layers, such as lamellar porous carbon stacks and expanded porous graphene films, to allow folding hundreds of times.^[^ ^25^, ^26^, ^27^ ^]^ The assembly strategy is also important for promoting flexibility. For instance, the assembly of graphene oxide platelets into honeycomb‐like macroporous structures can increase their mechanical flexibility.^[^ ^28^ ^]^ A carbon nanotube film with an aligned arrangement of few‐walled nanotubes can bear severe bending even folding for a hundred times.^[^ ^29^ ^]^ The current understandings of the relationship between structure and folding is limited and biased, just like the blind touching the elephant. Consequently, an integrated design of hierarchical structures to achieve super‐foldability has not been proposed and implemented.

After millions of years of evolution, nature has endowed organisms with rich structures and functions that provide remarkable inspiration for developing new materials.^[^ ^30^, ^31^, ^32^ ^]^ For example, cuit silkworm cocoons have super‐flexible features after reeling cocoon processing. Moreover, the *Mimosa* leaf possesses reversible and scatheless foldable capability when subjected to external stimulus (Figure 1b,c; Figure S2a, Supporting Information). If the combined mimicking of the super‐flexible hierarchical structures of cuit cocoons and the scatheless‐folding composite interfaces of *Mimosa* leaves can be achieved, then a super‐foldable composite may be manufactured (Figure 1b–e; Figure S2, Supporting Information). Silkworms produce cocoons by spinning and cocooning, but the resultant raw cocoons are rigid and possess cross‐linked structures (Figure S3a,b, Supporting Information). However, a facile reeling cocoon process can transform rigid cocoons into cuit cocoons with excellent foldable features. In this process, structural changes including unfastened junctions, slidable fibers, separable layers, and compressible networks are generated (Figure S3c,d, Supporting Information). Our further study for the first time revealed that these structural changes could result in intelligent deformation according to the bending degree, and form a layered nanofiber network “***ε***” structure at the crease under true‐folding, which performs a key function in super‐foldable performance (Figure 1e; Figure S4 and Note S2, Supporting Information). Under normal conditions, *Mimosa* leaves are open; however, they rapidly fold upon stimulation.^[^ ^33^ ^]^ Such a sensitive change is due to the important role of the pulvinus, which is composed of a vascular tissue as the core and parenchyma tissue formed by thin‐walled cells as the shell. Under normal conditions, parenchymal cells on both sides are filled with water. Once a stimulus occurs, one side of the parenchymal cells loses water and shrinks, resulting in leaf folding (Figure 1c). The adjustable intervals due to shrinking and stable interfaces ensure the intactness of cells. To quantitatively verify the role of the aforementioned biological structures during folding, mechanical simulations were implemented using three extracted hierarchies (Figure 1f–m). At the 3D assembly hierarchy, the stresses of biomimetic separable‐layer structures and ordinary block plate structures during folding are calculated. The former can disperse the stress to each assembly hierarchy through *ε* deformation. The distributed stress is considerably less than that of the latter, which is concentrated at the crease (Figure 1f,g). In the 2D layer hierarchy, the bending stress in biomimetic non‐crosslinked and crosslinked layer models was simulated. The results indicated that crosslinked structures rapidly boosted stress until fracture with increasing flexure, whereas biomimetic non‐crosslinked structures could effectively disperse the stress through fiber sliding (Figure 1h,i). At the 1D hierarchy of composite fibers, the bending stress distributions in three composite structure models, i.e., biomimetic fiber/cones, fiber/pillars, and fiber/dense‐wrapping, were simulated (Figure 1j–m). The simulation shows that the biomimetic fiber/cone has a better stress dispersion. The maximum stresses in the other two composite fibers reached 1.3 and 4.5 times that of the fiber/cone under the same degree of bending(Figure 1j). Based on the above analysis, a biomimetic fiber /cone‐array self‐adaptive web structure is proposed to develop super‐foldable composite.

To prove the feasibility of this biomimetic design strategy and verify the prospects of super‐foldable electrodes, FeOOH, which has multiple functions in energy, sensor and catalysis,^[^ ^34^, ^35^, ^36^, ^37^ ^]^ is selected for carbon nanofiber loading to fabricate C‐fiber/FeOOH‐nanocones web nanocomposites. Notably, their structures, functions, and preparation processes were bioinspired (Figure S2, Supporting Information). Specifically, electrospinning is applied to mimic the silkworm spinning and cocooning processes to obtain polymer webs. In‐situ gradient‐temperature carbonization, followed by liquid deposition, is applied to simulate the reeling cocoon process and biological super‐foldable structures to acquire the fluffy composites.^[^ ^38^, ^39^ ^]^ Accordingly, composites that are conductive and super‐foldable are produced by the biomimetic process along with artificial control. By combining the biomimetic techniques in terms of process and function, the super‐foldable C‐web/FeOOH‐nanocone material (SFCFe) was successfully prepared. This material can readily bear more than 100 000 times repeated true‐folding without microstructural damage and electrochemical property changes—certainly a breakthrough advancement in flexible electrode materials.

## 2 Result and Discussion

### 2.1 Structural Characterizations of SFCFe

**Figure 2:**
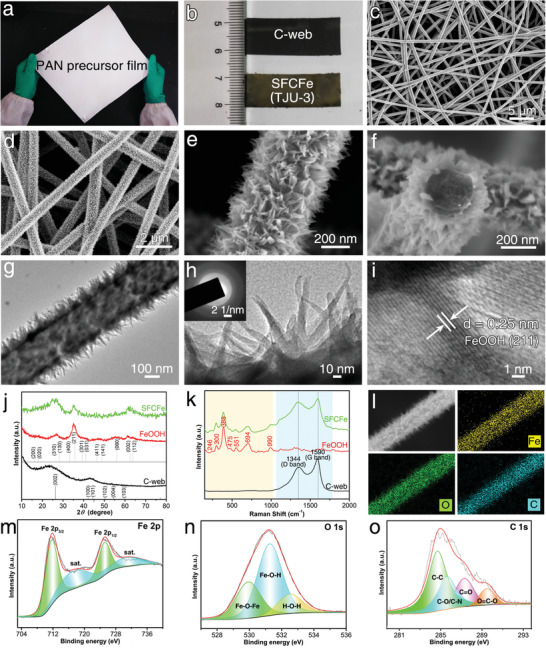
Characterizations of SFCFe. a,b) Optical photographs of PAN film, C‐web, and SFCFe. c–f) SEM images. g,h) TEM images (inset: SAED image). i) HRTEM image. j) XRD patterns. k) Raman spectra. l) EDS mapping images. m–o) XPS high‐resolution spectra of Fe 2p (m), O 1s (n), and C 1s (o).

The macro and micromorphologies of structures from polymer precursors to the final composite products are displayed in **Figure** **2** and Figure S5 in the Supporting Information. The C‐web substrates are first prepared through biomimetic electrospinning and in situ gradient‐temperature carbonization, causing the precursors change color from white to black (Figure 2a,b). The gradient‐temperature carbonization involves two processes: preoxidation in air for precursor stability and further carbonization in N_2_. The former removes adsorbed water and resident solvent, and starts cyclization and dehydrogenation reactions of PAN. Thus heat‐resistant ladder structures with well‐maintained morphology are produced, which enables morphology retention in the following high‐temperature carbonization. The two combined processes simultaneously achieve four changes including junction unfastening, layer separation, network unloosening, and pore generation similar to reeling cocoon process, and enable the successful preparation of conductive super‐flexible C‐web substrates like cuit cocoon structures (Figures S6 and S7, Supporting Information). During the subsequent mild liquid deposition, the desired biomimetic composite structures of FeOOH nanocones inlaid on carbon nanofiber surface form through mild‐temperature protection, morphology control, and time adjustment. It proceeds according to the following reactions

(1) $$\mathrm{CO}{\left(NH_{2}\right)}_{2}+H_{2}O\to 2NH_{3}+CO_{2}$$

(2) $$NH_{3}+H_{2}O\to N{H_{4}}^{+}+OH^{-}$$

(3) $$Fe^{3+}+3OH^{-}\to \mathrm{Fe}{\left(\mathrm{OH}\right)}_{3}$$

(4) $$\mathrm{Fe}{\left(\mathrm{OH}\right)}_{3}\to \mathrm{FeOOH}+H_{2}O$$

Scanning electron microscopy (SEM) observations show that the obtained SFCFe has hierarchical layer network structures woven by uniform straight nanofibers covered with fuzzy substances; the nanofiber diameter is ≈400 nm, and the mesh is several microns in size (Figure 2c,d; Figure S8, Supporting Information). Surface substances were found capable of passing through the mesh and growing firmly on each fiber inside the film because of surface hydrophilicity from pretreatment, contributing to improvement of electrochemical properties (Figure S9, Supporting Information). Enlarged SEM and transmission electron microscopy (TEM) images indicate that fuzzy fibers are coaxial structures composed of vertically grown FeOOH shells on carbon nanofiber cores (Figure 2e–g). Each FeOOH shell is cone‐like in morphology mimicking the interface structures of *Mimosa* leaf, which enables scatheless folding (Figure 2h). Its length and bottom diameters are ≈100 and ≈20 nm, respectively. Further structural characterizations revealed its polycrystalline structures and good crystallinity as determined from diffraction rings in its selected area electron diffraction (SAED) image and clear lattice spacing of 0.25 nm in its high‐resolution transmission electron microscopy (HRTEM) image, respectively, benefitting fast electrochemical reaction (Figure 2h,i). Moreover, the grown density and loaded mass of FeOOH nanocones can be well controlled by simply adjusting the reaction time, contributing to the optimization toward both super‐foldable and high‐electrochemical properties.

Condition experiments indicate that reaction conditions such as temperature, time, and additive, will influence the FeOOH morphologies and composite constructions, and thus the mechanical flexibility. For temperatures of 30 °C, the reaction nearly does not start (Figure S10, Supporting Information). With the temperature increase, the reaction begins to accelerate. The temperature of 40 °C only produces large aggregated FeOOH clusters distributed unevenly on fiber surface, and their further growth causes crosslinks of nanofibers and layers, which are unfavorable for flexibility (Figure S11, Supporting Information). While the higher temperature of 50 °C leads to the uniform and dense growth of FeOOH nanocones at 6 h, but afterwards they will become overcrowded and stuck the networks, which is disadvantageous for folding (Figure S12, Supporting Information). When the temperature reaches 60 °C, a too fast reaction speed makes the FeOOH deviate from cone‐like structures and become more crowded, resulting in the composites stiff and brittle like the raw cocoons (Figures S13 and S14, Supporting Information). Seen from above results, the optimal condition for desired biomimetic structures is reaction at 50 °C for 6 h, because at that time the FeOOH have cone‐like morphologies, and distribute uniformly on fiber surface, and do not cause crosslinks of nanofibers and layers. As for the Na_2_SO_4_ additive, comparison experiments show that its addition as a strong electrolyte can accelerate the crystal nucleation and growth (Figure S15, Supporting Information). Meanwhile, it also controls the cone‐like morphology of FeOOH, because the SO_4_^2−^ ions with cone‐like regular tetrahedral configurations may be able to adsorb on specific crystal planes and induce their anisotropic growth (Figure S16, Supporting Information).

Their structures were further characterized by X‐ray diffraction (XRD) and Raman. The successful formation of these composite structures is proved by the appearance of broadened diffraction peaks of both graphite (JCPDS no. 41–1487) and *α*‐FeOOH (JCPDS no. 18–0639) in the XRD patterns, which may be caused by the partially crystalline structures of carbon and the nanosize effect of FeOOH, respectively (Figure 2j).^[^ ^40^ ^]^ The Raman spectrum of the C‐web shows two peaks at 1344 and 1590 cm^−1^ in the blue region, corresponding to the D and G bands of typical carbon materials. A higher ratio of G band to D band (I_G_/I_D_ = 1.3) indicates the relatively high graphitized structures of the C‐web (Figure 2k). Seven peaks, i.e., 246, 300, 388, 475, 551, 694, and 990 cm^−1^, appear in the Raman spectrum of pure FeOOH in the yellow region; these values well conform with those reports regarding *α*‐FeOOH. The SFCFe's Raman spectrum displays peaks of both substances, indicating their successful combination. Their elemental composition and binding states are measured by energy dispersive X‐ray spectroscopy (EDS) and X‐ray photoelectron spectroscopy (XPS). Both the EDS and XPS results show the existence of Fe, C, and O elements that are homogeneously distributed on the nanofiber (Figure 2l–o; Figures S17–S20, Supporting Information). The curve fit of Fe 2p spectrum exhibits two major peaks, 711.8 and 725.2 eV, corresponding to Fe 2p_3/2_ and Fe 2p_1/2_ with shake‐up satellites at ≈718.3 and 731.7 eV, which characterize Fe^3+^ in FeOOH (Figure 2m).^[^ ^41^ ^]^ The peaks of O 1s spectrum are centered at ≈530.2, 531.7, and 532.8 eV, corresponding to the oxygen element in the Fe–O–Fe, Fe–O–H, and H–O–H bonds, respectively (Figure 2n). All the above characterizations indicate that the desired composite structures have been successfully obtained.

### 2.2 Super‐Foldability of SFCFe

**Figure 3:**
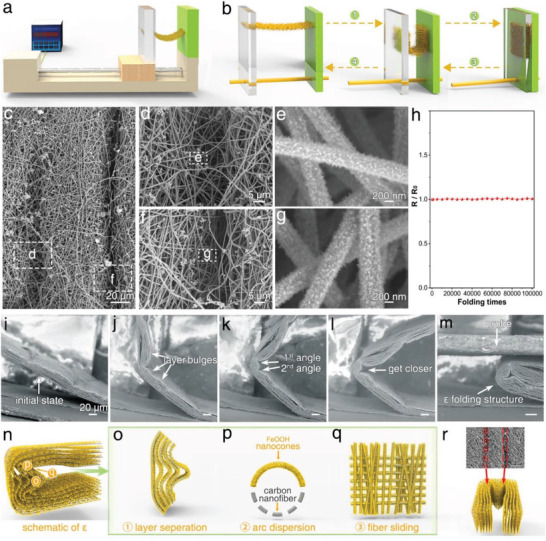
Super‐Foldable characterizations of SFCFe. a) Schematic of repeated true‐folding test on a folding machine. b) Schematic of a complete true‐folding process. c–g) SEM images of SFCFe after 100 000 times folding. h) Conductivity change curves during 100 000 times folding. i–m) Typical states during real‐time SEM observation of folding process. n) Schematic of “***ε***”‐like structures at crease at 180° true‐folding. o–r) Enlargement and analysis of “***ε***” structures in (n).

The folding properties and corresponding structural analysis are shown in **Figure** **3**. Figure 3a shows our self‐made folding machine that can automatically conduct folding tests and record folding times. More importantly, it does not only satisfy the true‐folding operation up to the standard, but also ensure folding force uniformity and folding position accuracy (Figure S21 and Movie S1, Supporting Information). In folding studies, the three requisites above must all be satisfied simultaneously; otherwise, reporting the material as foldable and their folding times is pointless. Figure 3b shows the unfolded, bent, and fully folded states of SFCFe on the folding machine. An optimized super‐foldable SFCFe is eventually obtained through persistent exploration for materials and repeated folding tests. Amazingly, it can sustain more than 100 000 times of true‐folding without microstructure damage or conductivity changes (Figure 3c–h). However, two microtraces that are invisible to the naked eye appear; they result from nanofiber slide and density change for improving stress dispersion and structural self‐protection (Figure 3c). Further, the observation and analysis of creases indicate that not only the composite‐nanofibers have no fracture or damage, but also the FeOOH‐nanocones have no detachment from the nanofiber surface, demonstrating the feasibility of inlaying or assembling microdevices on super‐foldable substrates (Figure 3d–g). The microstructures of SFCFe at different folding times have also been tracked, and results show that they are intact (Figure S22, Supporting Information). By contrast, the C‐web/FeOOH composites with overcrowded FeOOH loading generate structural damage after one time folding, and the accumulated damage results in material fracture after limited folding times (Figures S23–S25, Supporting Information). This indicates that their its folding capacity is history‐independent, and they have no fatigue accumulation; consequently, they can be folded numerous times without limit. Besides the above arbitrary folding times, the SFCFe also features an arbitrary directional folding ability, as demonstrated by their different folding forms (Figure S26, Supporting Information). In addition, the SFCFe sample is tested by severely twisting and rolling it around an ultrathin rod with a 0.5‐mm radius. After removing the applied external force, the sample quickly recovers its original shape and shows no structural fractures (Figure S27, Supporting Information). Such excellent twisting and rolling properties play an auxiliary role toward attaining super‐foldable performance. The foregoing demonstrates that the super‐foldable property of arbitrarily repeated folding has been realized for the SFCFe. This also means that stable electrochemical performance can be ensured when the SFCFe is subjected to repeated folding.

### 2.3 Working Mechanism of SFCFe

To reveal its super‐foldable mechanism, we have also developed a dynamic folding observation system for the real‐time tracking of microstructural changes during folding. Five typical dynamic states are captured and displayed in Figure 3i–m_._ Initially, the SFCFe was flat (Figure 3i). As the bending degree is increased, stress among adjacent layers is gradually generated, and the layers begin to separate, forming wavy bulges to disperse stress (Figure 3j). With further bending, stress concentrates at the crease, forming two folding arcs to disperse the stress (Figure 3k,l). When the SFCFe is folded to 180°, a smooth “***ε***” structure eventually forms, as shown in Figure 3m. Such constructions are the true root of the super‐foldable property; the structures are virtually the same as those in a folded cuit cocoon. The real‐time unfolding process shows that the “***ε***” gradually disappears, and the initial flat state of structures is virtually recovered (Figure S28, Supporting Information). The entire folding and unfolding processes of SFCFe exhibit a self‐adaptive stress dispersion behavior through intelligent deformation according to the bending degree. In general, the above dynamic observation is extremely vivid and reliable for folding mechanism studies; it is possibly the first real‐time SEM record of the folding process worldwide.

By analyzing the “***ε***” folding structure, the stress dispersion functions can be examined in detail (Figure 3n; Figure S29, Supporting Information). The “***ε***” structure contains three typical regions: bulged layers, dispersed arcs, and folded traces. The bulged layers are caused by layer separation, which can disperse the stress along the layer direction (Figure 3o). The two dispersed arcs with smooth shapes prevent stress concentrations (Figure 3p; Figure S23g–i, Supporting Information). During arc formation, the inner space of the crease is redistributed, and the layers at the arcs are compressed. As a result, the stress at the top of the two arcs is dispersed. As for the two folded traces, they are just located at the same position as the two dispersed arcs and extend toward the inside, which can disperse the stress in the thickness directions by the sliding of nanofibers (Figure 3q,r). In summary, the formation of the ***ε*** structure is accompanied by inner space redistribution, which can effectively disperse stress at various levels and directions and eventually realize the super‐foldable property.

### 2.4 SFCFe as Super‐Foldable Anode Materials

**Figure 4:**
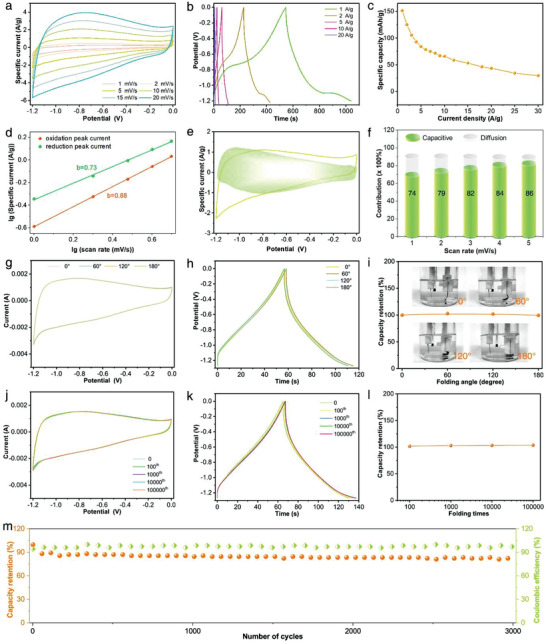
Electrochemical performance of SFCFe as super‐foldable anode materials. a–c) CV, GCD, Cs curves of SFCFe. d) Relationship between peak currents and scan rates. e) Capacitive and diffusion‐controlled contributions for charge storage at 5 mV s^−1^. f) Normalized contribution ratios of capacitive and diffusion‐controlled capacities at different scan rates. g–i) CV, GCD, and capacity retention curves at different bent angles. j–l) CV, GCD, and capacity retention curves during 100 000 times repeated folding. m) Cycling property of SFCFe.

The electrochemical properties of SFCFe composites were studied using a three‐electrode system in 6 m KOH (**Figure** **4**). First, the electrochemical properties of the C/FeOOH composites obtained at different synthesis times (3, 6, and 9 h) were compared. Based on the comparison of geometric areas of cyclic voltammetry (CV) curves and discharge times of galvanostatic charge‐discharge (GCD) curves, the specific capacities of composites were observed to display a volcanic‐shape trend with increasing synthesis time, achieving the peak value at 6 h (Figure S30a,b, Supporting Information). The results of electrochemical impedance spectroscopy (EIS) show that the charge transfer resistance (*R*_ct_) is positively related to the loading mass of FeOOH. However, excessive loading is not advantageous because overdense FeOOH (such as composites obtained at 9 h) limits the transfer of electrons/ions and availability of atoms (Figure S30c, Supporting Information). The SFCFe obtained at 6 h was subsequently selected for examining the electrochemical properties in detail. Its CV curves deviate from the rectangle with a larger potential window of 0 to −1.2 V, which indicates the SFCFe's partly faradaic energy storage behavior (Figure S31–S33, Supporting Information). The similar shapes of these curves from 2 to 20 mV s^−1^ indicate satisfactory conductivity (Figure 4a). Consistent with CV results, the GCD curves are non‐linear with plateaus, also indicating the partly faradaic energy storage feature of SFCFe (Figure 4b). The calculation results show the SFCFe's high specific capacity and satisfactory rate capability: its maximum capacity (152 mAh g^−1^ at 1 A g^−1^) is 7, 3, and 1.4 times higher than that of C‐web (22 mAh g^−1^), C/FeOOH at 3 h (48 mAh g^−1^), and C/FeOOH at 9 h (111 mAh g^−1^), respectively (Figure S25, Supporting Information). It can be charged and discharged at a high current density exceeding 20 A g^−1^ and still maintained a specific capacity of 45 mAh g^−1^ at 20 A g^−1^ (Figure 4c). Reaction dynamics were further investigated to determine the origin of its outstanding electrochemical performance (Figure 4d–f). As shown in Figure 4d, for reduction and oxidation processes, the b values are 0.73 and 0.88, respectively, which means its current contains both surface capacitive and diffusion‐controlled processes. The calculated capacitive contribution values indicate a boost with the increase of scan rates, reaching 86% for the total charge storage at 5 mV s^−1^ (Figure 4e,f). Thus, the dominant capacitive contributions simultaneously result in high capacity and satisfactory rate capability. Compared with other materials reported in the literature, the SFCFe fabricated in this work exhibits the highest specific capacity among the flexible electrodes of FOOH composites. Moreover, foldable FeOOH electrodes have not been reported, not to mention the super‐foldable ones (Table S1, Supporting Information).

The super‐foldable properties of SFCFe have been demonstrated from aforementioned three aspects, i.e., of micrology, micromechanics, and electricity, and herein it will be further verified through electrochemistry. The tests are conducted by measuring electrochemical properties at different folding angles and folding times. On the one hand, the SFCFe is in situ bent to different angles, and their electrochemical properties are measured at corresponding angles. Results show that the CV and GCD curves are basically coincident and their corresponding capacities are nearly changeless at different bent angles, respectively (Figure 4g–i). On the other hand, the electrochemical properties of SFCFe are recorded during 100 000 times repeated folding. Remarkably, its CV and GCD curves are nearly overlapped, and capacities are almost the same throughout the repeated folding process, which is often difficult to achieve (Figure 4j–l). Besides, the cycling properties of the SFCFes after folding for 100 000 times are tested, and display a capacity retention of 84% after 3000 times (Figure 4m). The electrochemistry results obtained at the above two folding forms manifest our SFCFe can bear 100 000 times folding without structure damage or material detachment.

## 3 Conclusion

Introducing the self‐adaptive stress dispersion mechanism into the structure design, this work reports a universal approach to fabricating super‐foldable conductive electrode that can bear 100 000 time true‐folding without structure damage and conductivity degradation. The biomimetic strategies for both the preparation method and material structures were verified to be effective for achieving super‐foldable properties. Through the real‐time SEM observation of the folding process and their mechanical simulations, the relationship between biomimetic structures and super‐foldable performance is visually revealed. More importantly, a set of universal principles for constructing super‐foldable composite electrodes is identified as follows. (1) The electrode had better possess layered network structures in which nanofibers are slidable, and the layers are microseparable. (2) The electroactive substances on nanofibers should have special structures to cope with a certain bending. (3) Under a true‐folding state, the crease should be capable of forming “***ε***”‐like structures containing bulged layers, dispersed arcs, and slidable microtraces for 3D stress dispersion. In summary, the high‐performance electrode, exceptional folding properties, and revealed mechanism identify key materials and techniques for fabricating super‐foldable devices even assembled electronic equipment.

## Conflict of Interest

The authors declare no conflict of interest.

## Supporting information

Supporting Information

Supplemental Movie 1

## Data Availability

The data that supports the findings of this study are available in the supplementary material of this article.
