# Supplementary material for: Bioinspired Nanocomposites with Self‐Adaptive Stress Dispersion for Super‐Foldable Electrodes
Source: Adv Sci (Weinh). 2021 Nov 17;9(3):2103714. doi: 10.1002/advs.202103714 (PMC8787393; doi:10.1002/advs.202103714)
Supplement: Supplementary file 1 — Supporting Information [file ADVS-9-2103714-s001.pdf]

WILEY-VCH

© 2021 Wiley-VCH GmbH

ADVANCED SCIENCE
Open Access

Supporting Information

for *Adv. Sci.*, DOI: 10.1002/advs.202103714

Bioinspired Nanocomposites with Self-adaptive Stress Dispersion for Super-Foldable Electrodes

Guangtao Zan, Tong Wu, Zhenlei Zhang, Jing Li, Junchen Zhou, Feng Zhu, Hanxing Chen, Ming Wen, Xiuchun Yang, Xiaojun Peng, Jun Chen,* and Qingsheng Wu**

Supporting Information

Bioinspired Nanocomposites with Self-adaptive Stress Dispersion for Super-Foldable Electrodes

*Guangtao Zan, Tong Wu, * Zhenlei Zhang, Jing Li. Junchen Zhou, Feng Zhu, Hanxing Chen, Ming Wen, Xiuchun Yang, Xiaojun Peng, Jun Chen,* and Qingsheng Wu**

This PDF file includes:

Experimental section

Figures S1 to S33

Table S1

Notes S1 and S2

Caption for Videos S1

Other Supplementary Materials for this manuscript include the following:

Videos S1

Experimental section

Preparation of the carbon web substrates. The super-flexible carbon web substrates were prepared by improved electrospinning /carbonization method. First, a certain polyacrylonitrile (PAN) (Mw=150,000) powder was dissolved in DMF to form a homogeneous solution with 10% concentration. Then, the above solution was put into a 20 mL plastic syringe which was connected to a stainless-steel needle with 0.6 mm internal diameter. The electrospinning of PAN was operated under the electrospinning voltages of 12.5 kV, the solution flow rate of 0.5 mL h^{-1}, and collecting distance of 16 cm, and the temperature and humidity were respectively controlled at ~25 ℃ and ~35% RH. Afterwards, the electrospun PAN film was peeled off from the Al foil collector, and dried at 60 °C in an oven before the following carbonization transformation process. During the carbonization process in a tube furnace, the PAN film underwent gradient temperature heating in air for stabilizing. It was firstly heated at 105 °C for 30 min with a heating rate of 2 °C min^{-1}, and then heated at 160 °C for 30 min with a rate of 1 °C min^{-1}, and further heated at 270 °C for 2 h with a rate of 1 °C min^{-1}. Subsequently, the stabilized products were carbonized at 800 °C for 2 h in N_2 atmosphere with a heating rate of 2 °C min^{-1} to obtain free-standing carbon substrates.

Preparation of super-foldable C-web/FeOOH-nanocone (SFCFe). In a typical synthesis, the above carbon substrates was firstly pretreated by soaking in the 50 mL aqueous solution containing 20 mL HNO_3 for 12h, which favors inward diffusion of solution into carbon web and increases the interfacial combination between nanofibers and functional matters. Then 0.1 g of $FeCl_3 \cdot 6H_2O$, 0.25 g of Na_2SO_4 and 0.2 g of $CO(NH_2)_2$ were dissolved in 50 mL of distilled water to form homogenous reaction solution. Afterwards, five pieces of the pretreated carbon substrates were added into the reaction solution. The solution was heated at 50°C for 6 hours. After cooled to room temperature naturally, the products were separated, washed and dried at 60 °C overnight to obtain super-foldable SFCFe anode materials. For the inlaid quantity control of FeOOH nanocones, the reaction times are set as

3h and 9h with other synthesis conditions are the same as above. To explore the role of Na_2SO_4, the reaction solution for depositing FeOOH was prepared without Na_2SO_4, and other reaction conditions were the same as the above SFCFe preparation process.

Materials characterization. Field emission scanning electron microscopy (FE-SEM, Hitachi S-4800) and transmission electron microscopy (TEM, JEOL JEM-2100) were used to observe the morphologies and microstructures of the products. The EDS system attached to the FE-SEM was applied to measure the elemental contents and distributions of products. X-ray photoelectron spectroscopy (XPS, AXIS Ultra DLD) was applied for surveying the bonding states of surface elements. An X-ray diffractometer (Bruker D8 Focus) with Cu Kα radiation (λ= 0.15418 nm) was used for analyzing crystal structures of products at preset voltage of 40 kV and current of 40 mA. An Invia confocal Raman system was exploited to obtain the Raman spectra of products at the laser wavelength of 514 nm. The electrical resistance variations of products at different folding cycles were in-situ measured on the folding machine using a digital multimeter. The water contact angles of the samples were measured using a CA100B from Shanghai Innuo Precision Instruments Co., Ltd at 25 °C.

Flexibility tests. The foldable performance of products was evaluated by different types of true-folding operations. Manual folding deformation along different directions was conducted to judge their arbitrary foldability. Cyclic folding tests of products on folding machine were operated, and their microstructures were observed off-line by FE-SEM to evaluate their foldable performance. The Real-time SEM observation of the folding process was performed on a Phenom G2 Pro SEM with a folding stage. The twisting and rolling tests of products (4×40 mm^2) were carried out around a stainless steel rod with a very small radius of 0.5 mm.

Mechanical simulations. Finite element method was applied for the mechanical simulations of material folding process from three different structural levels. At 1D nanofiber level, three composite nanofiber models of different FeOOH morphologies (nanocones, nanopillars, and densely wrapped

structures) loaded on carbon nanofiber were constructed. And the stress distributions of the above three fiber models were calculated by three-point bending simulations to the same bending degree, and the maximum stress values are obtained from the simulated results. At 2D layer level, we built two representative layer models, which are crosslinked layer and non-crosslinked layer, and calculated their stress distributions during bending. In this process, the carbon/FeOOH composite nanofibers were equivalently simplified as simple nanofibers. At 3D body level, two kinds of 3D folding structures, the super-foldable stereostructure and brittle stereostructure, were established and simulated

Electrochemical measurements. Electrochemical properties were measured on a CHI660E electrochemical workstation (Shanghai Chenhua Instruments Co.). The electrochemical performance of the freestanding C-web/FeOOH-nanocones materials was evaluated in a three-electrode system in 6M KOH aqueous electrolyte at room temperature, and they were directly used as working electrode and their a platinum foil and an Ag/AgCl electrode acted as the counter electrode and reference electrode, respectively. For the investigation of the electrochemical property during cyclic folding, the electrode materials were taken down from electrode holder, and folded for different times on the folding machine, and placed back on electrode holder for tests. The specific capacities (C_s, mAh/g) were calculated from galvanostatic charge-discharge (GCD) curves by the formula of $C_s = I\Delta t/m$, where I is the constant current (mA), m is the mass (g) of electrode material, and Δt is the discharge time (h) during the discharge process.

Supplementary Note 1

Metals are made up of metallic bonds that are non-directional, thus most of them are flexible and can be bent or even folded for a certain times. After all, metallic bonds belong to chemical bonds which are short-range force, so they can't resist large bending deformation. As a result, when they undergo 180° true-folding, their structures will be damaged. Eventually, material fracture will occur due to cumulative damage caused by repeated folding. Conductive polymers consist of conjugated Π bonds, which have the nature of double bonds and thus are more rigid than single bonds, so they can hardly bear large bending deformation, not to mention repeated true-folding. For the abundant carbon materials, the flexible conductive ones mainly include graphene, carbon nanotubes and partially graphited carbon. Monolayer graphene is an ultrathin plane structure and shows some flexibility. While its constructions of sp2 hybrid conjugate Π bonds have the property of double bonds, so single-layered graphene also can't sustain repeated true-folding. With regard to a single-walled carbon nanotube, it is actually equivalent to the rolled monolayer graphene, and of course can't bear numerously repeated true-folding. Thus it can be concluded that none of the intrinsic conductive materials can sustain numerous repeated true-folding when they directly cope with folding using chemical bonds due to the limit of short-range force of chemical bonds.

Supplementary Note 2

As a source of inspiration for innovation, the cuit silkworm cocoon with super-flexible feature is investigated in detail from processing to structures. The starting materials for preparing cuit cocoon, raw cocoon, are actually stiff and difficult to be folded, but they become flexible and foldable just after simply boiling in alkaline aqueous (reeling cocoon process). The SEM observation shows that the completely folded cuit cocoon can form a 3D "**ε**"-like structure at the crease due to redistribution of space, which means some parts are compressed and some are loosened around the crease. The "**ε**"-like structures contains bulged layers and two dispersed arcs, and those structural changes can effectively disperse stress through avoiding the formation of 0° folding angle. To unraveling the mysteries of above property variation, the material constructions before and after the reeling process are investigated. Results indicate the cross-linked network structures of raw cocoon, whose fibers are wrapped by the sericin and glued tightly at their intersections. While for cuit cocoon, their intersections are completely unfastened, and the fibers becomes porous and fluffy. The above analysis give us new inspiration to obtain similar structures. The silkworm constructs cocoon by

spinning, and it reminds us of the polymer nanofibers electrospinning, which can produce abundant transformable hierarchical structures, such as adjustable pores, detachable intersections, separable layers, and nimble nanofibers. However, the electrospinning polymer with satisfying transformation can only be obtained by parameter optimization, just like that the structure of raw cocoon requires the control of silkworm spinning. Even though obtaining such materials, it is a pity they are still not conductive. At that aspect, the precisely controlled carbonization process provides an efficient way to simultaneously realize material conductivity and structural maintaining.

Supplementary Figures

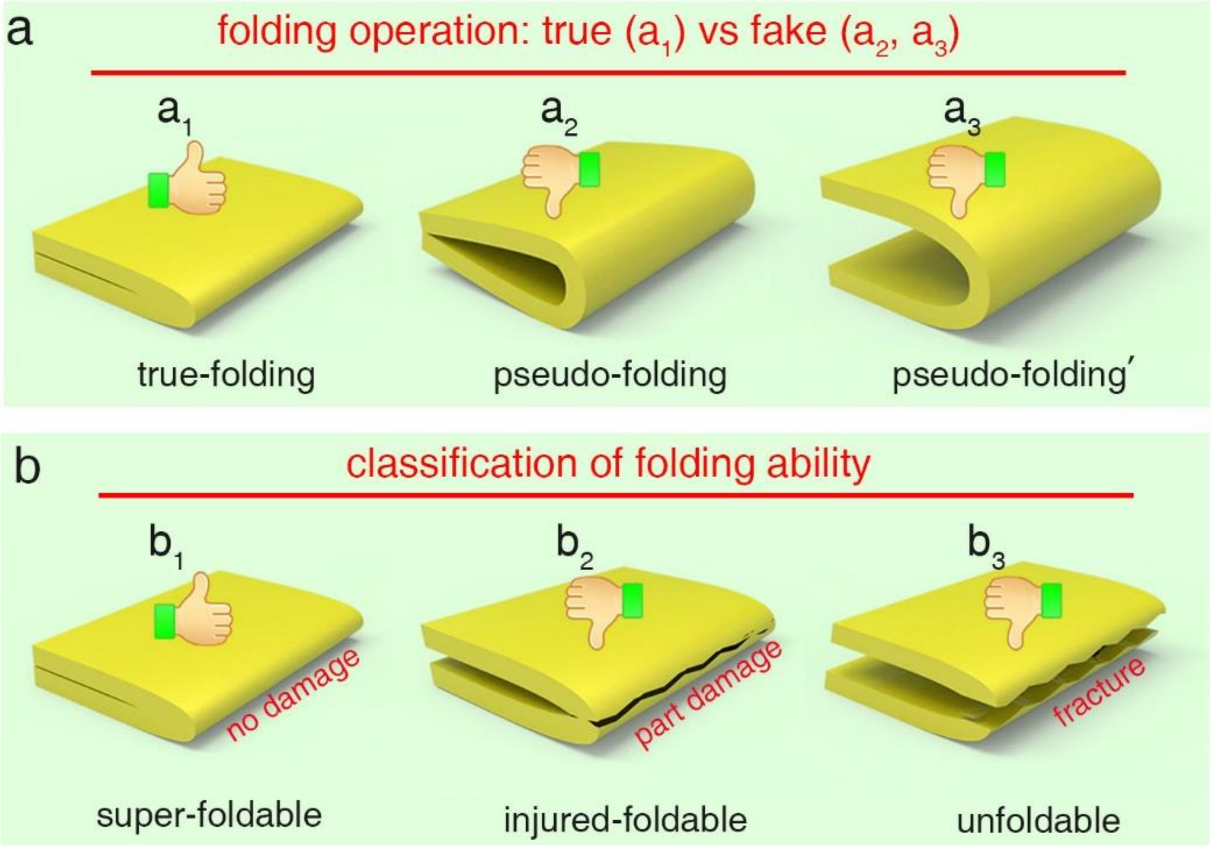

Figure S1. Concepts in folding study. (a) Different folding operations. (b) Classification of folding ability.

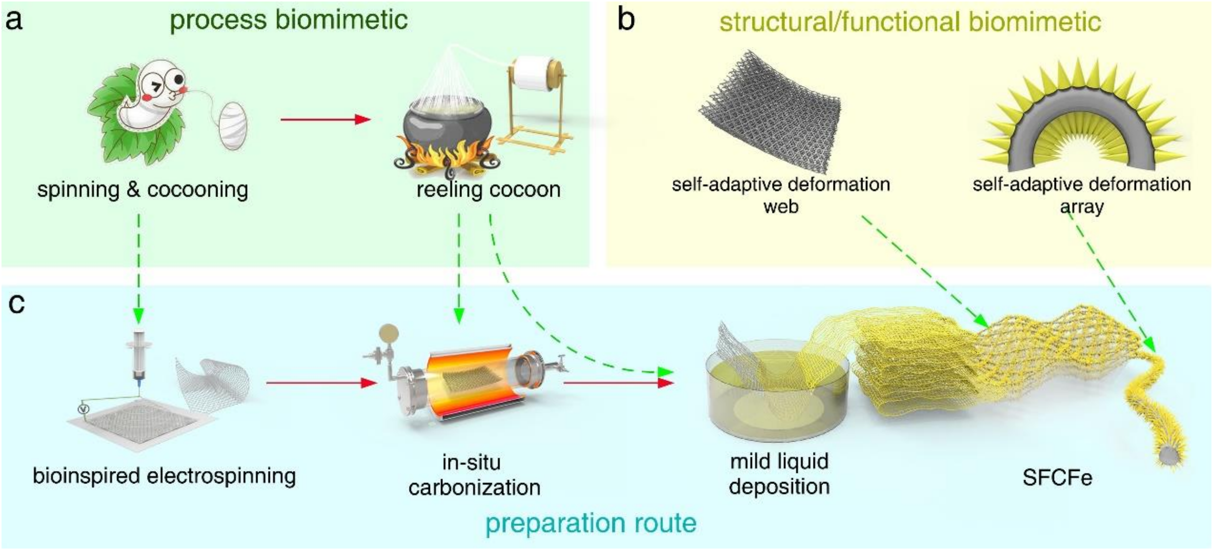

Figure S2. Congruent relationship between preparation route and process/ function biomimetic synthesis

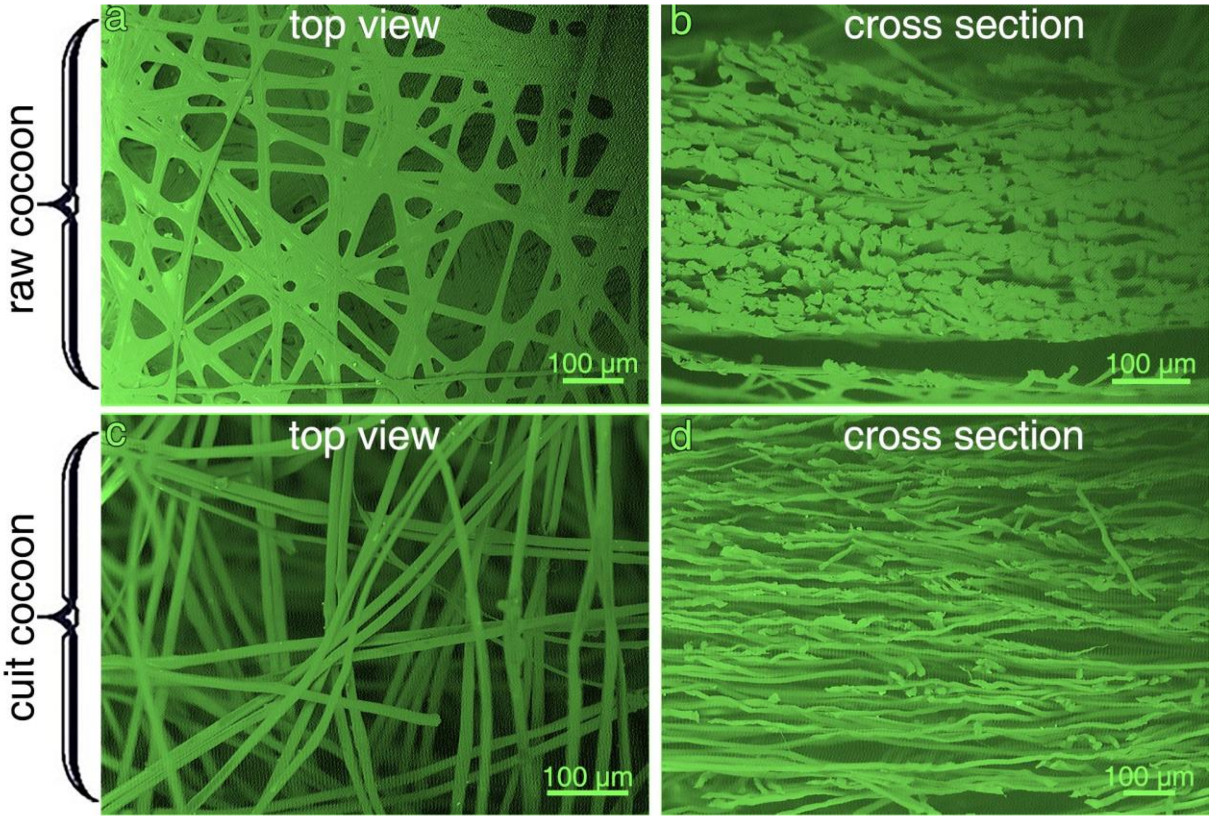

Figure S3. Microstructures of raw and treated cocoon at different views. (a, b) SEM images of raw cocoon. (c, d) SEM images of cuit cocoon.

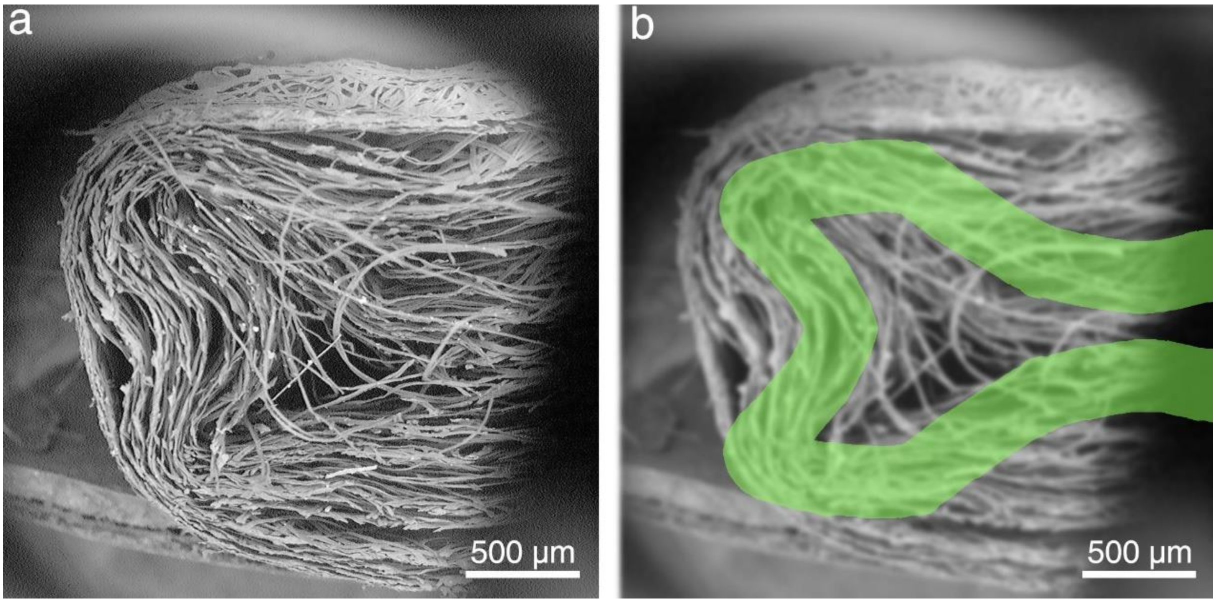

Figure S4. SEM images of crease of folded cuit cocoon.

SEM image shows a “ε”-like structures of the folded part.

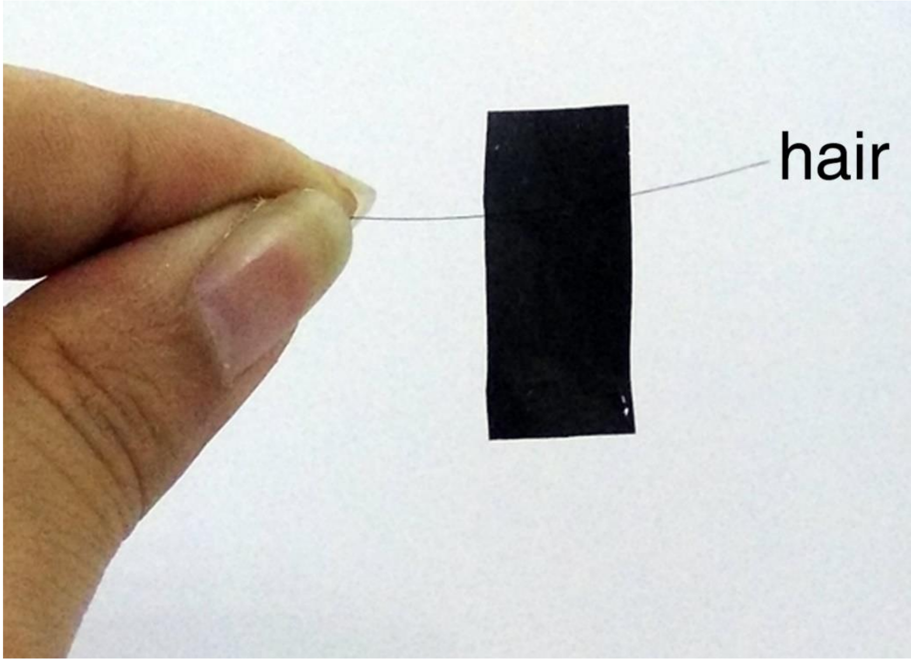

Figure S5. A piece of C-web substrate adsorbed on a hair showing its ultralight feature.

Result shows that the C-web substrate can hang on a hair through electrostatic adherence, while the hair has no bending deformantion, which indicates its ultralight feature.

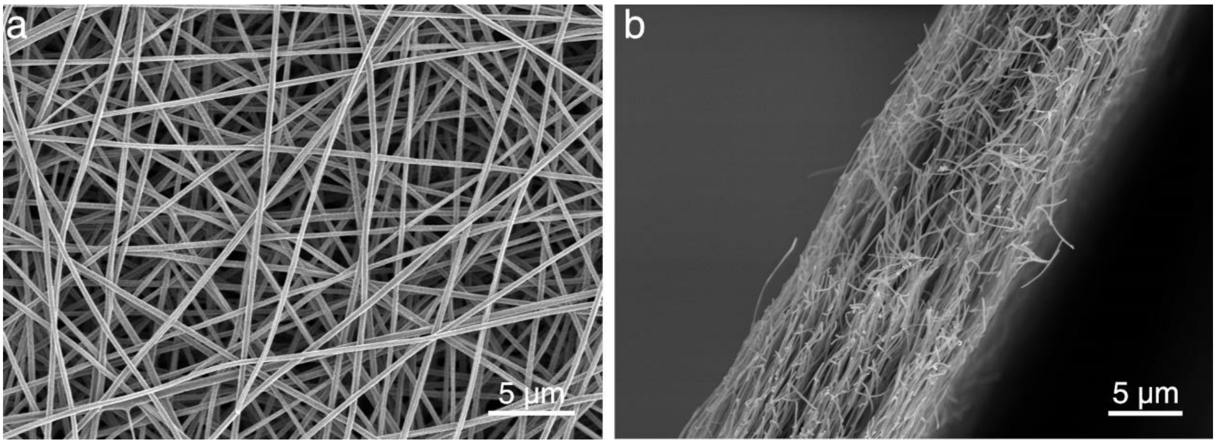

Figure S6. SEM images of super-flexible C-web substrate at top view (a) and cross section (b).

Results indicate the C-web substrate has network structures stacked by layers of carbon nanofibers. Those nanofibers are straight and smooth with large aspect ratio. Notably, they are stacked in order and have no adhesion between each other.

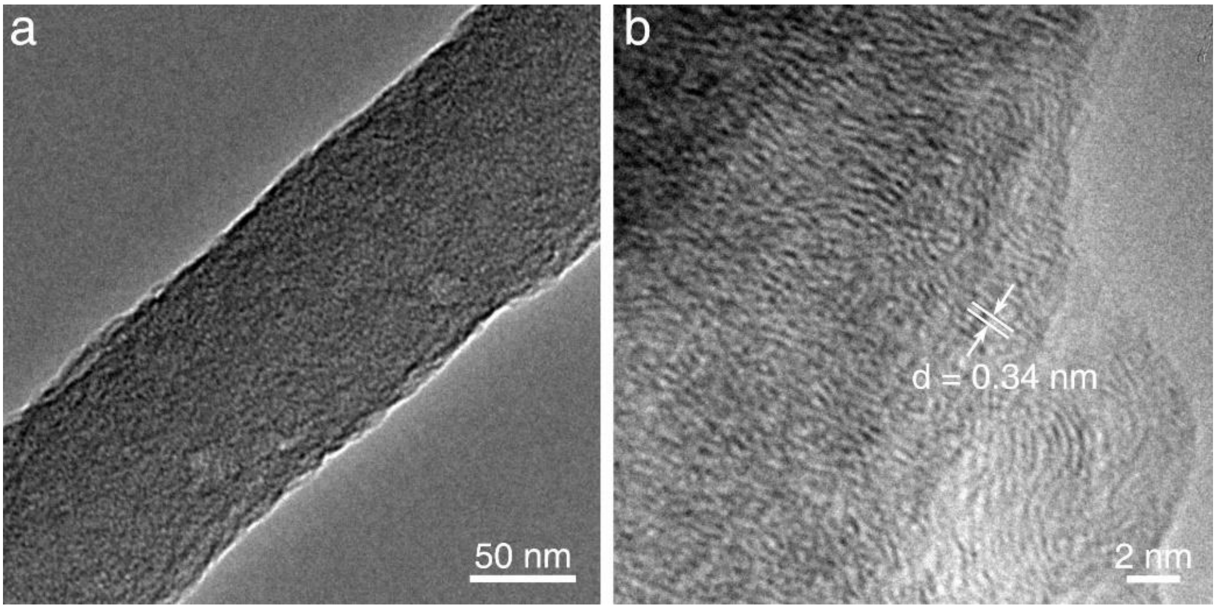

Figure S7. TEM and HRTEM images of super-flexible C-web substrate.

Results show that the nanofiber is porous. And the interplanar spacing of 0.34 nm can be indexed to (002) crystal face of graphite, indicating its partially graphitized structure. Such porous and partially graphitized structures endow the carbon nanofibers with both high flexibility and good conductivity.

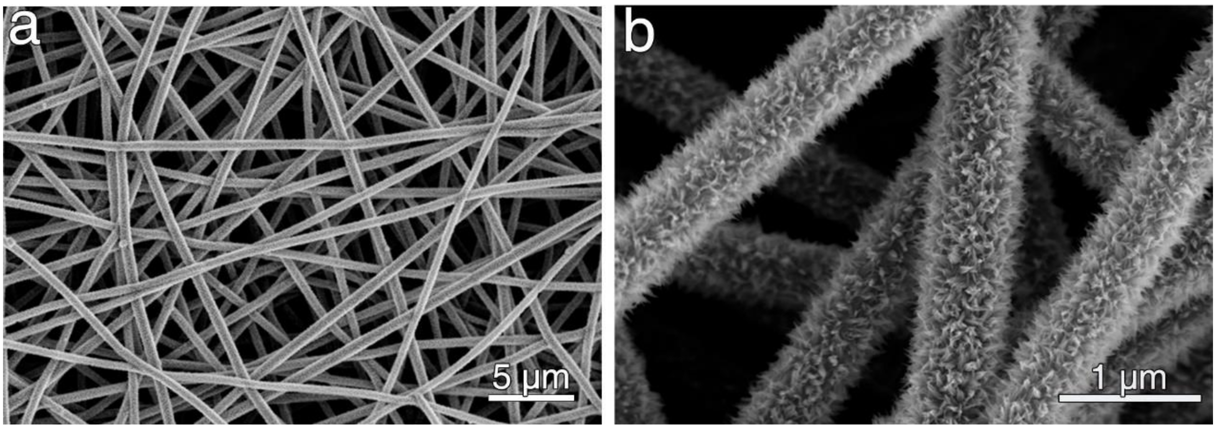

Figure S8. SEM images of SFCFe at different magnifications.

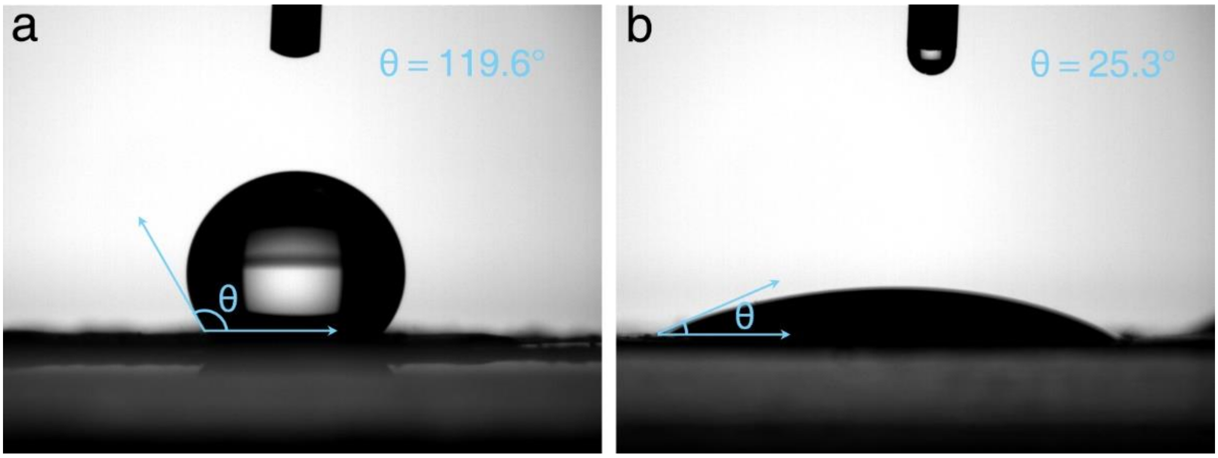

Figure S9. Contact angles of raw (a) and pretreated (b) carbon substrates at room temperature.

Results indicate that the raw carbon substrate is hydrophobic with a contact angle of 119.6° , while the pretreated carbon substrate becomes hydrophilic with a contact angle of 25.3° , which may be due to the introduction of oxygen-containing functional groups on the fiber surface during pretreated process. The hydrophilic property makes the FeOOH pass through the mesh and grow on each fiber inside the film.

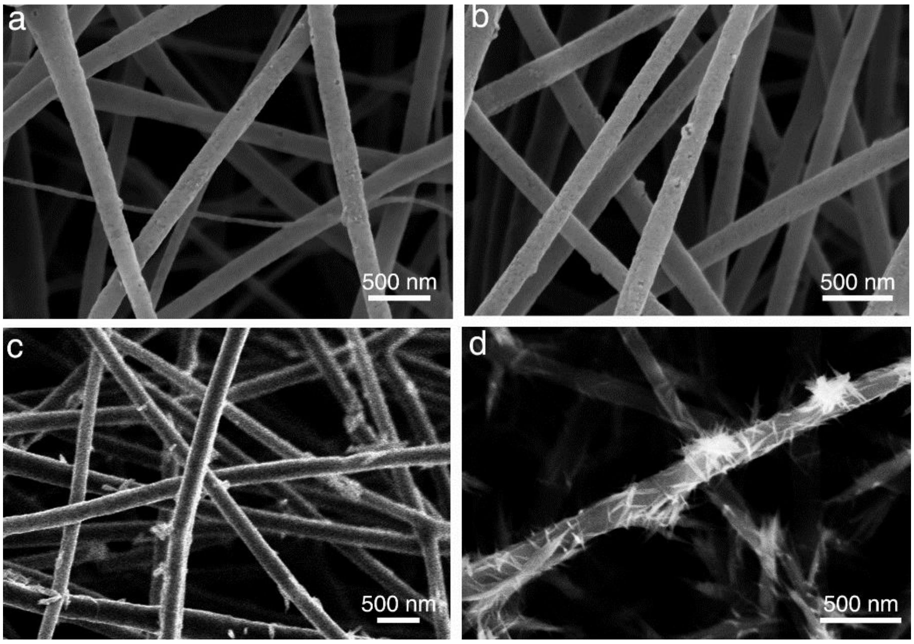

Figure S10. SEM images of C-web/FeOOH synthesized at 30℃with Na_2SO_4 for (a) 3h, (b) 6h, (c) 9h and (d) 12h.

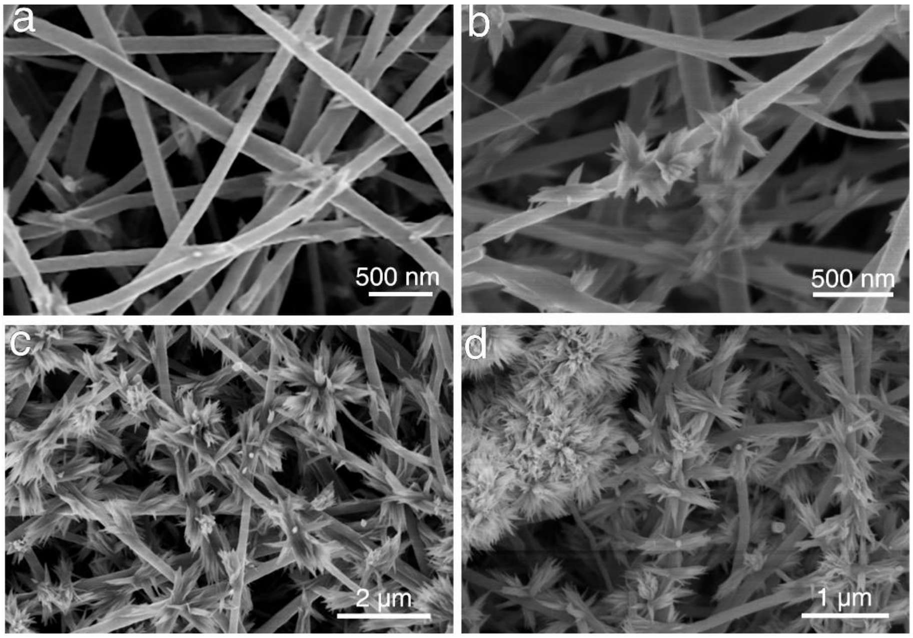

Figure S11. SEM images of C-web/FeOOH synthesized at 40℃with Na_2SO_4 for (a) 3h, (b) 6h, (c) 9h, and (d) 12h.

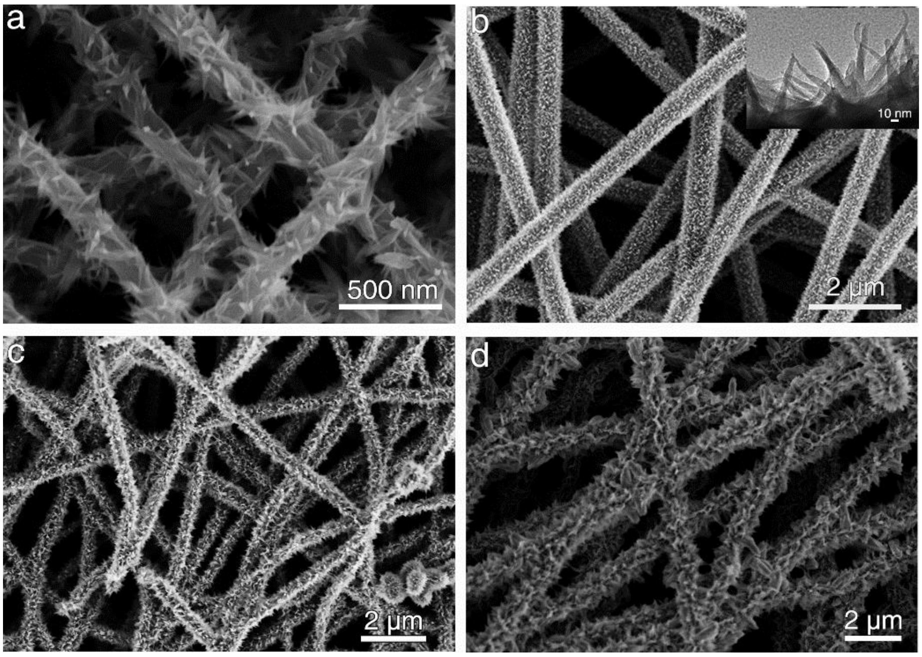

Figure S12. SEM images of C-web/FeOOH obtained at 50 °C with Na_2SO_4 for (a) 3h, (b) 6h, (c) 9h and (d) 12h.

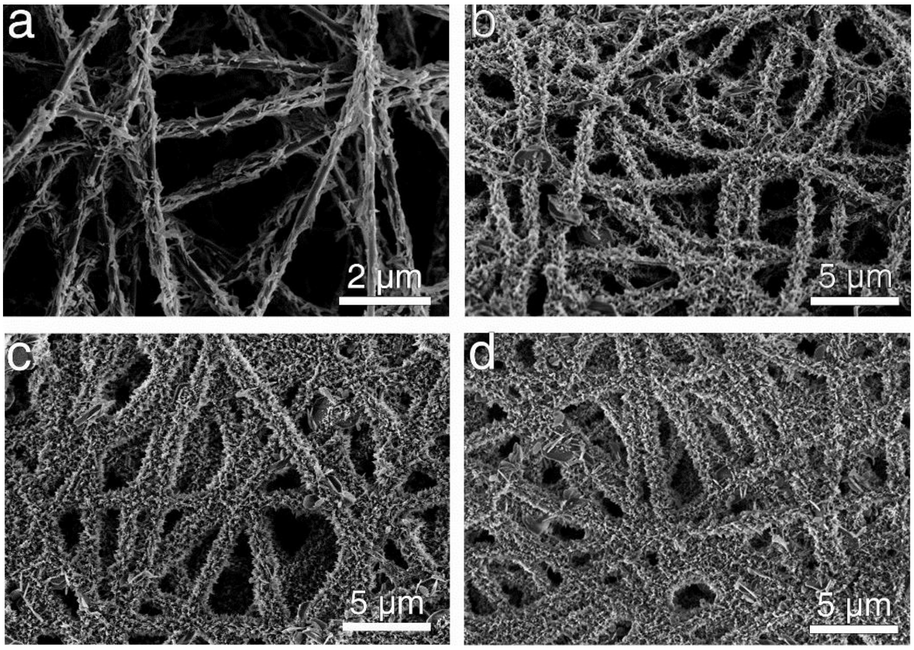

Figure S13. SEM images of C-web/FeOOH synthesized at 60℃with Na_2SO_4 for (a) 3h, (b) 6h, (c) 9h, and (d) 12h.

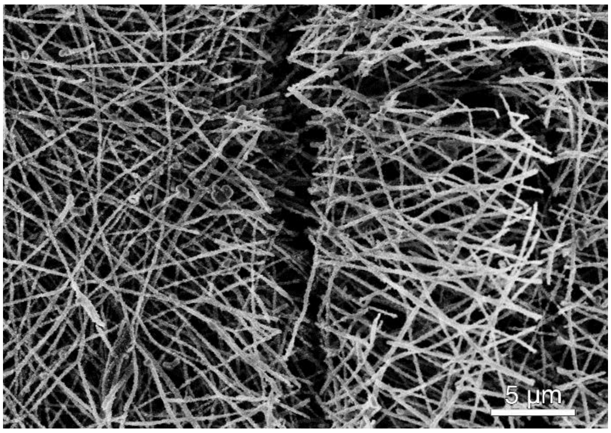

Figure S14. SEM images of C-web/FeOOH synthesized at 60℃for 6h with Na_2SO_4 after one-time folding

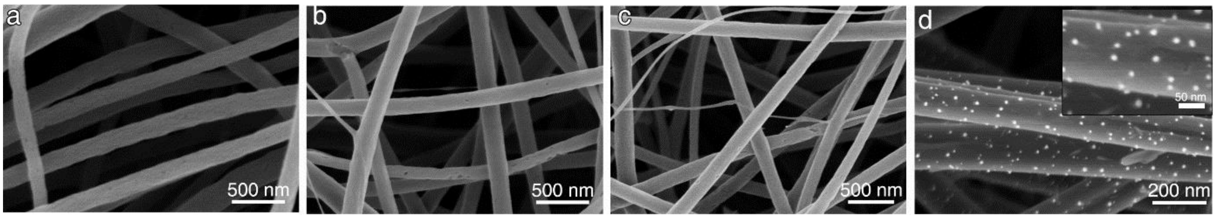

Figure S15. Influences of Na_2SO_4 on reaction. SEM images of C-web/FeOOH nanocomposites synthesized at 50 ℃without Na_2SO_4 at (a) 3h, (b) 6h, (c) 9h, and (d) 12h. Inset in (d): SEM image of enlarged FeOOH particles on nanofiber surface.

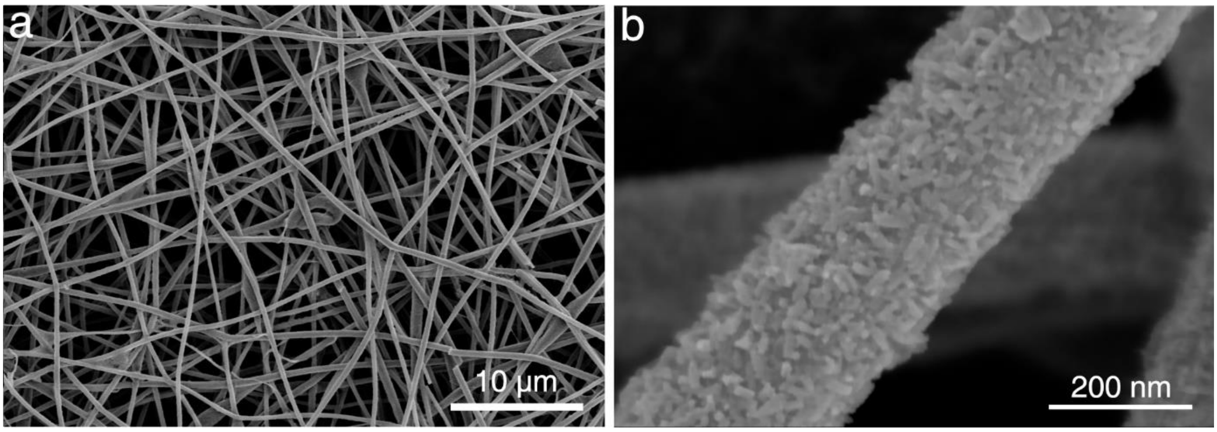

Figure S16. SEM images of C-web/FeOOH nanocomposites synthesized at 50 ℃for 72h without Na_2SO_4 at different magnifications.

The obtained products show irregular FeOOH particles covering on carbon nanofibers, indicating the important role of Na_2SO_4 for cone-like morphology control of FeOOH.

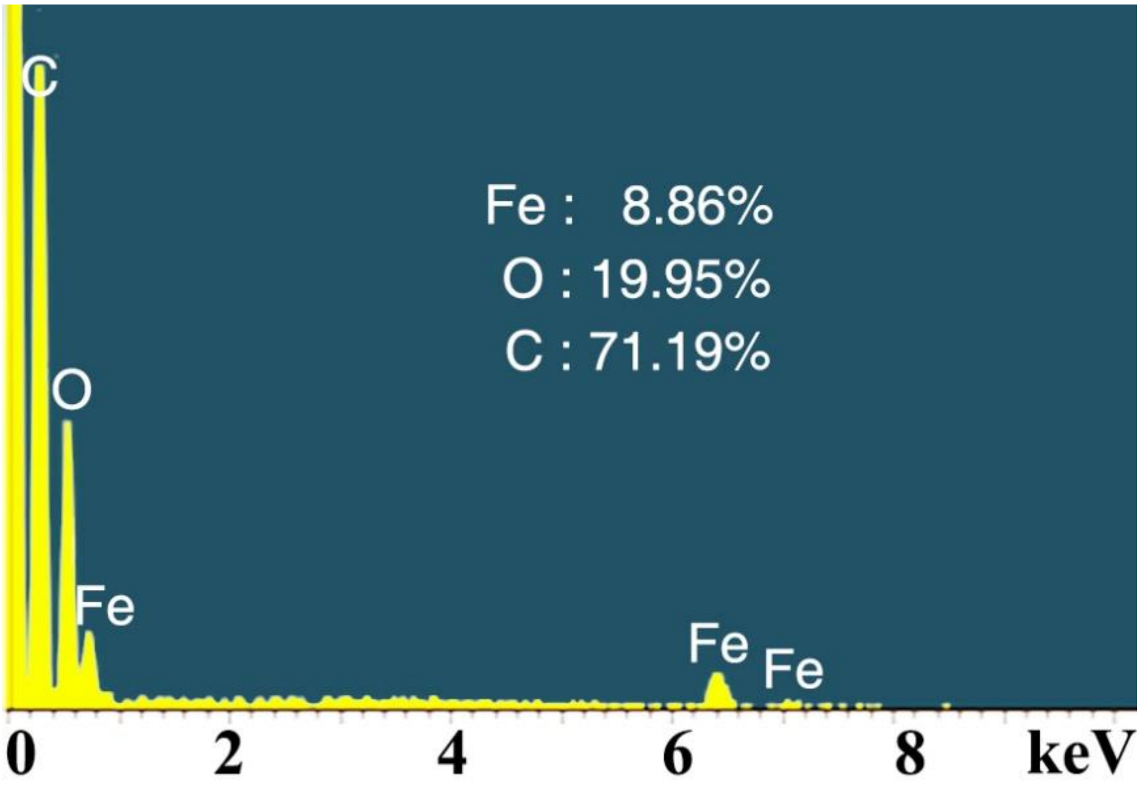

Figure S17. EDS spectrum of SFCFe

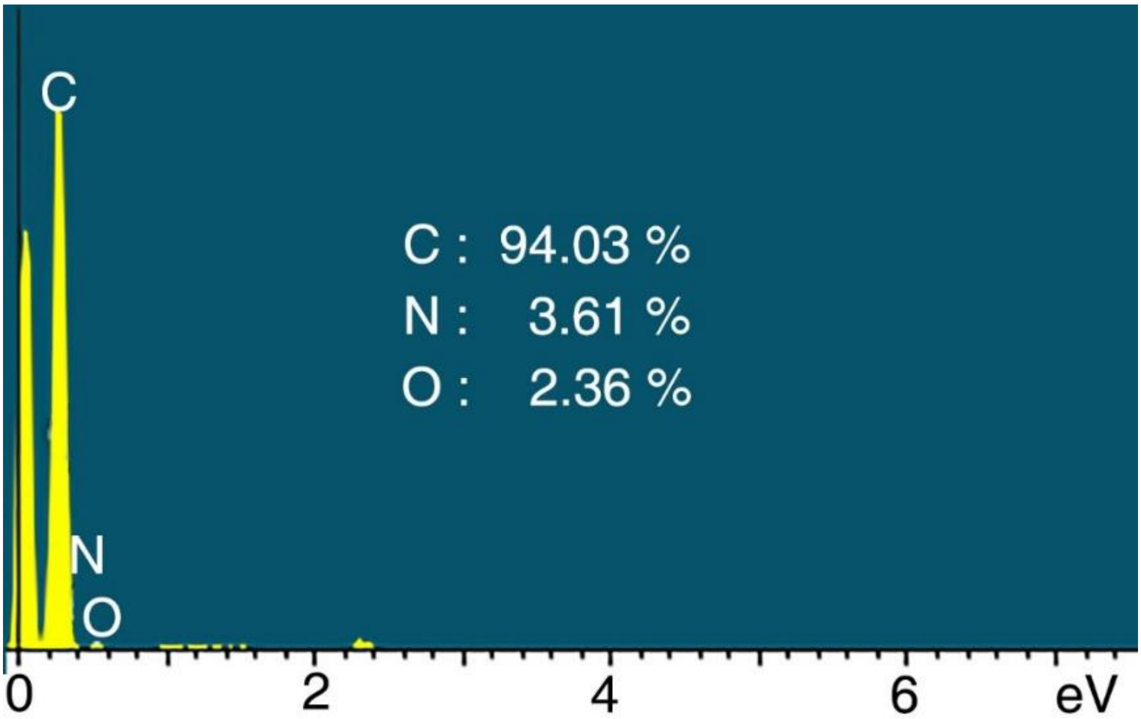

Figure S18. EDS spectrum of C-web substrate.

Result shows its components of massive C, slight N and O.

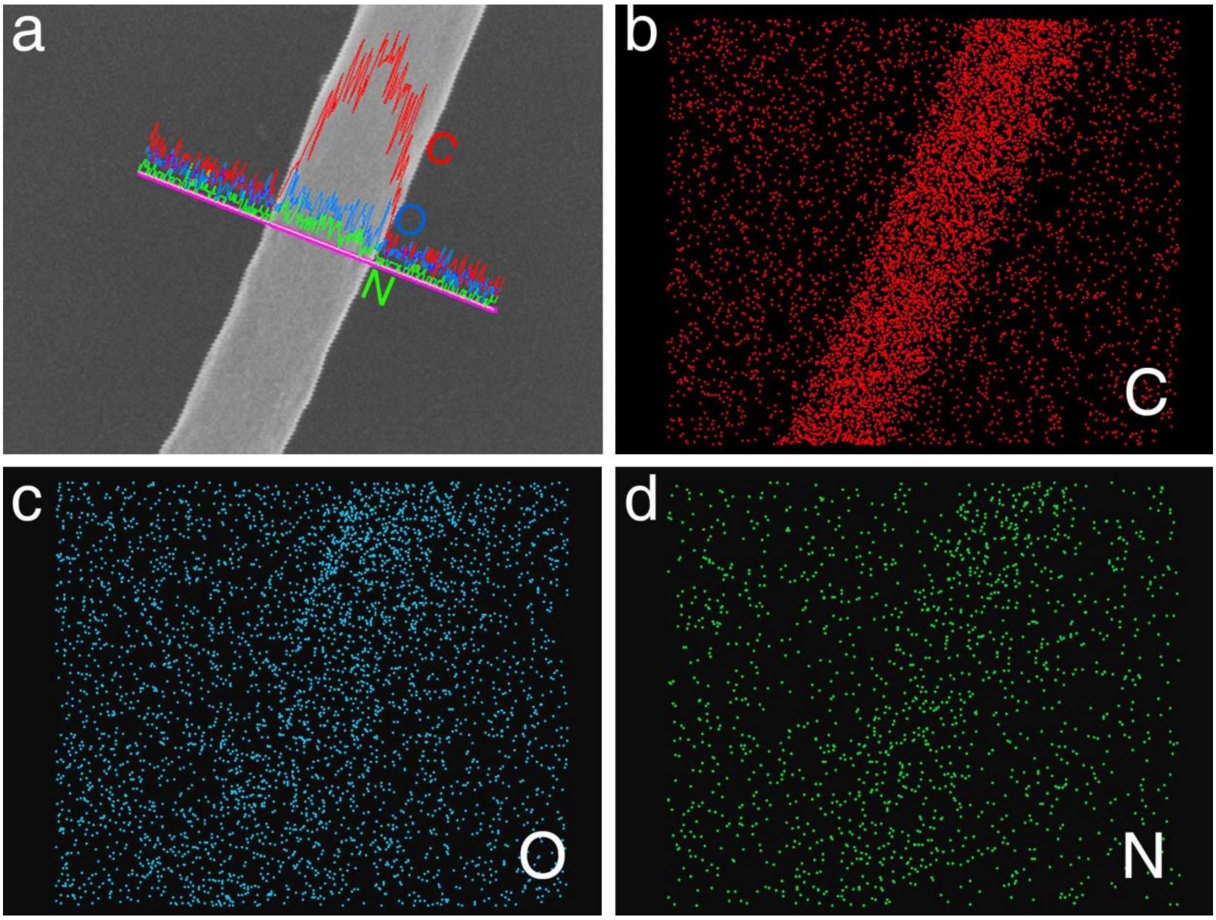

Figure S19. EDS line sweeping and elemental mapping spectra of C-web substrate.

Elemental concentrations along the pink sweeping line are consistent with the EDS spectrum. All the elements distribute uniformly in the nanofibers.

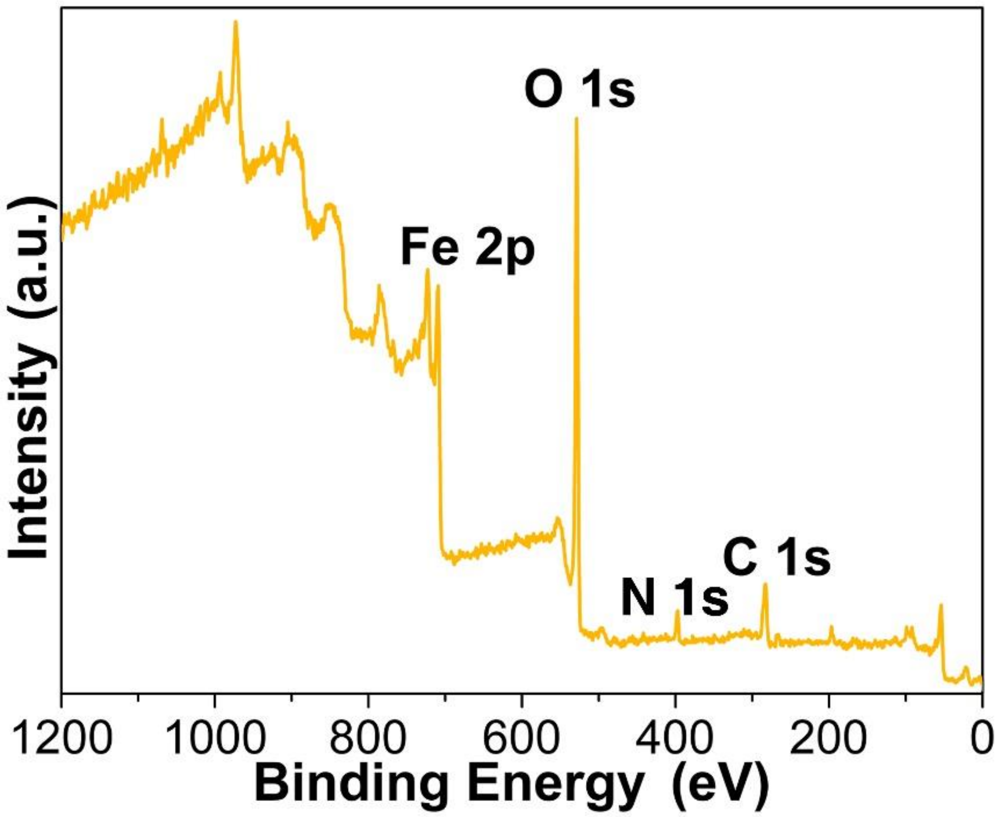

Figure S20. XPS survey spectrum of SFCFe.

XPS result also displays the existence of Fe, C, O, N, which is consistent with the EDS results.

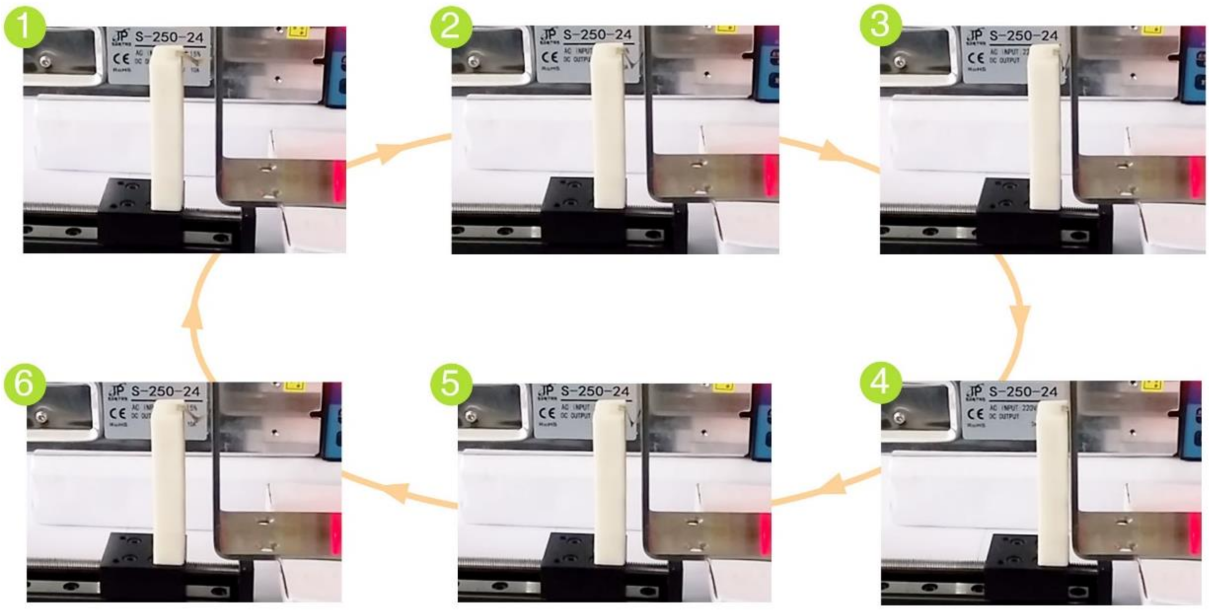

Figure S21. Typical statuses of SFCFe during one true-folding operation on the folding machine

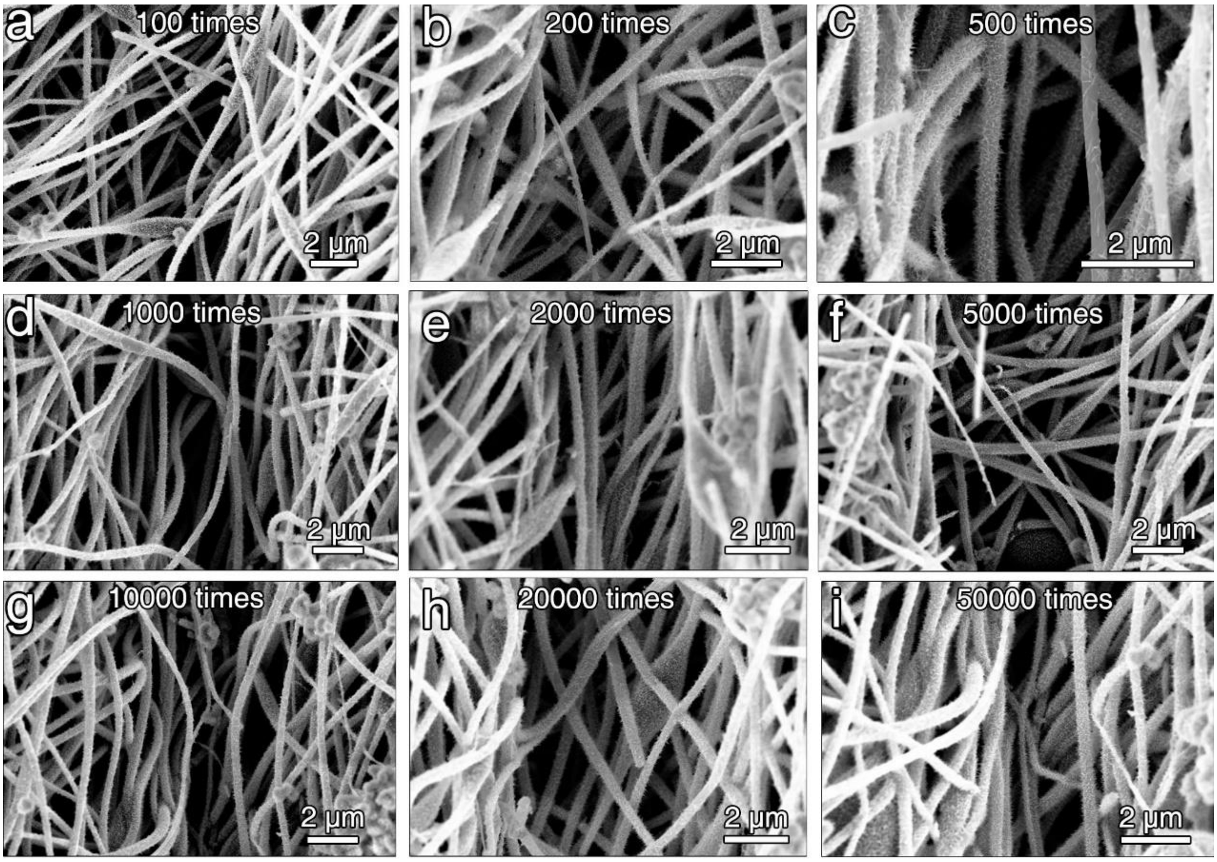

Figure S22. SEM images of inner side of SFCFe during 100,000 times true-folding

A similar situation arises that two micro-grooves appear for SFCFe samples of different times true-folding. Here only one micro-groove is enlarged for better observation for each sample. Results indicate that the SFCFe show little change and have no damage and detachment throughout the 100,000 times true-folding.

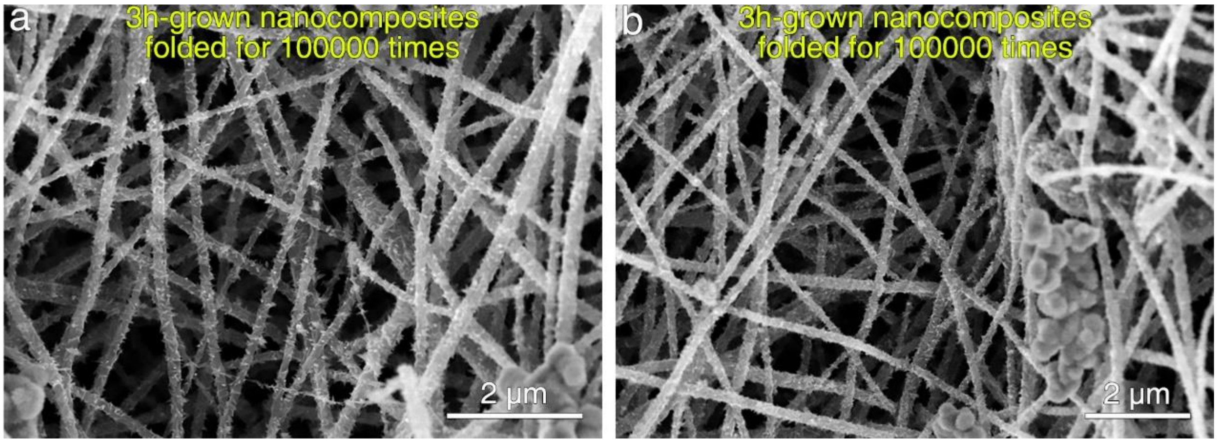

Figure S23. SEM images of two micro-grooves of C-web/FeOOH nanocone synthesized at 3h after cyclic true-folding for 100,000 times.

It shows that the products with lower loading amount and density of FeOOH also have super-foldable property, and they don't have microstructural damage or detachment.

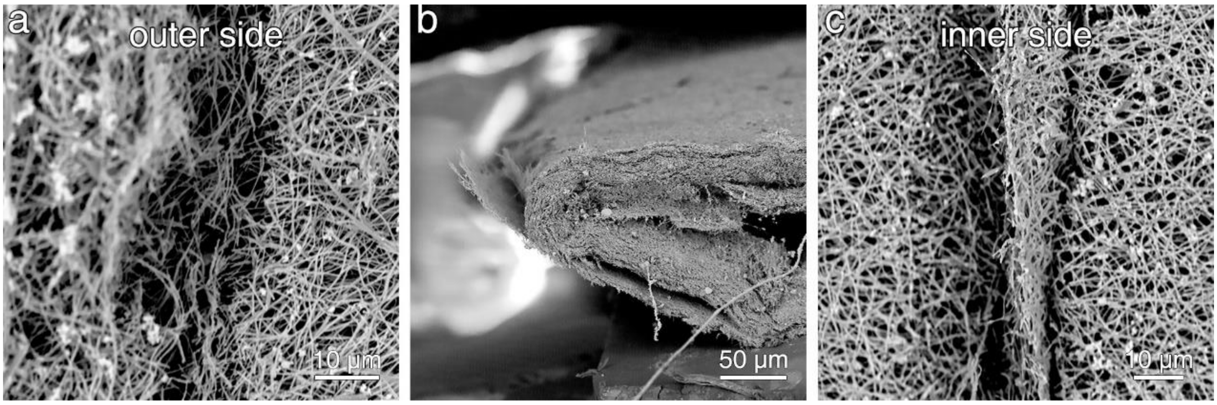

Figure S24. SEM images of C-web/FeOOH synthesized at 9h after one time folding operation, showing damaged structures.

Results indicate that too much loading of FeOOH nanocones on the C-web may result in the reduction of flexibility, and eventually the obtained composites are easily damaged even cracked once folded.

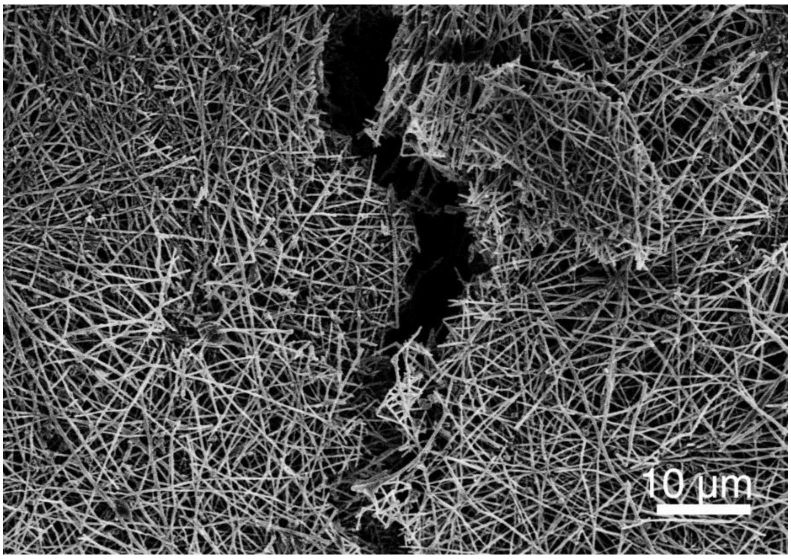

Figure S25. SEM image of fratured C-web/FeOOH synthesized at 12h after one time folding.

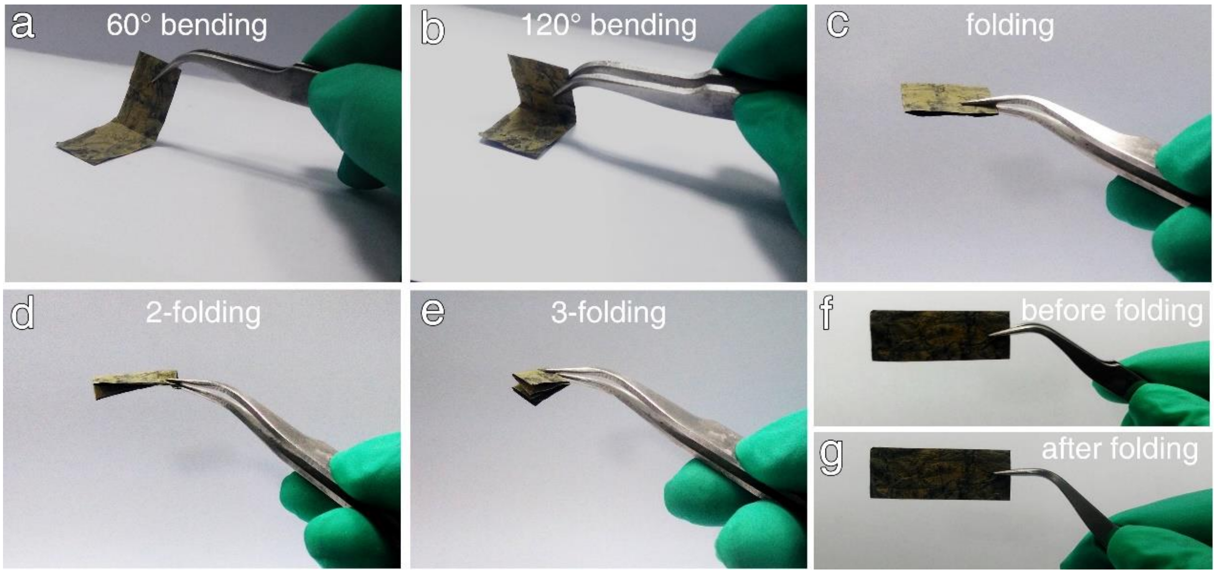

Figure S26. Various bending/folding tests of SFCFe. (a-e) Optical photographs of 60° bended, 120° bended, 1 folded, 2 folded and 3 folded SFCFe. (f, g) optical photographs of SFCFe before and after folding.

The SFCFe has no structure damage during the severe folding process and quickly recovers to its initial status once unfolded, indicating their outstanding undamaged foldability.

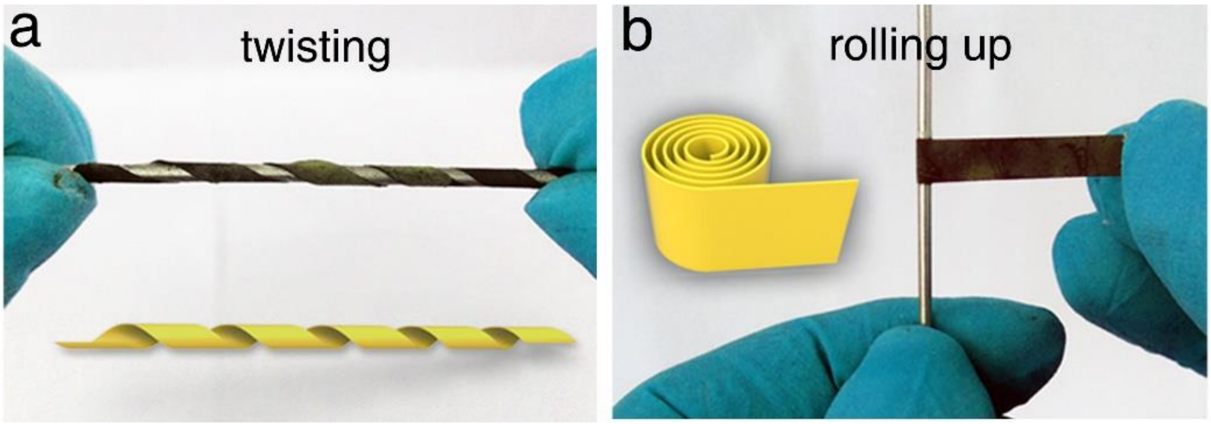

Figure S27. Twisting and rolling tests of SFCFe

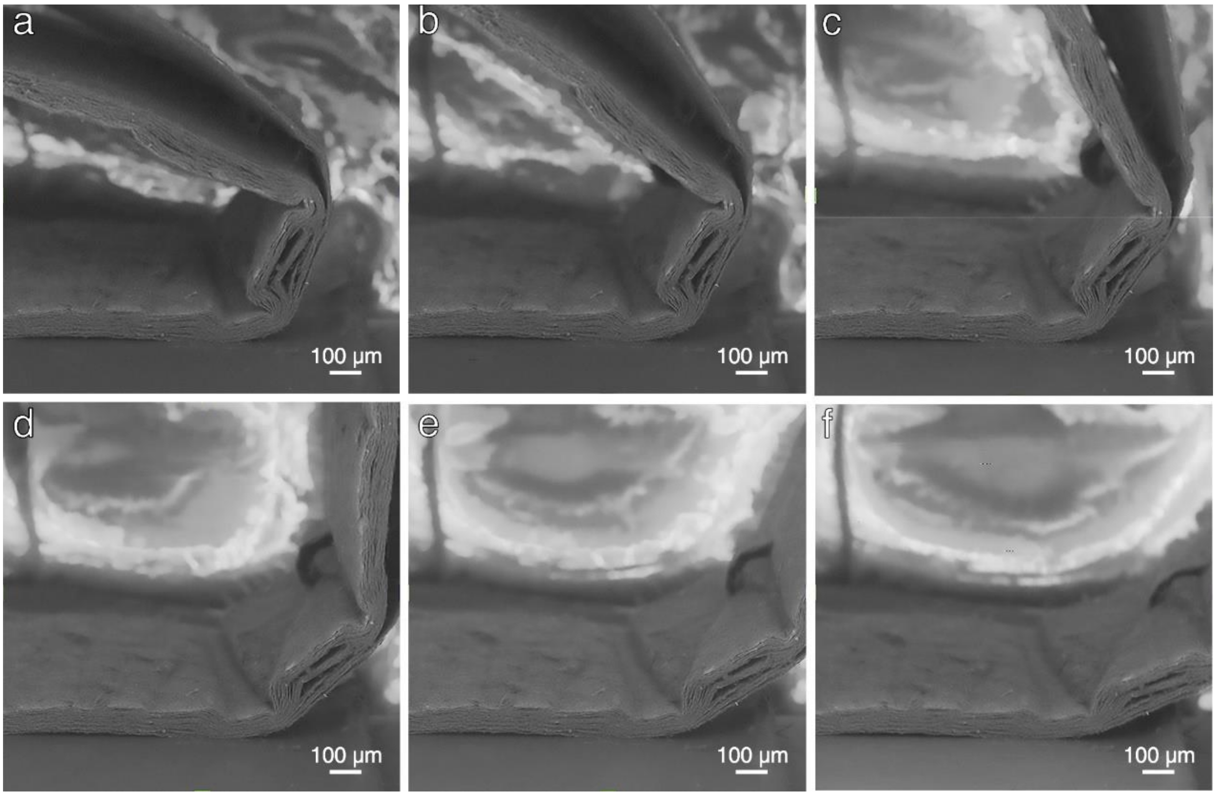

Figure S28. SEM images of real-time unfolding process of super-foldable SFCFe.

Results manifest that its unfolding process is just opposite to the folding process, and eventually, the SFCFe can nearly recover to its initial state. It is reasonable that the unfolded structures are not completely the same as their initial states, because the unfolded structures have the lowest energy and most stable state through nanofibers' sliding and adaptive adjustment in folding process.

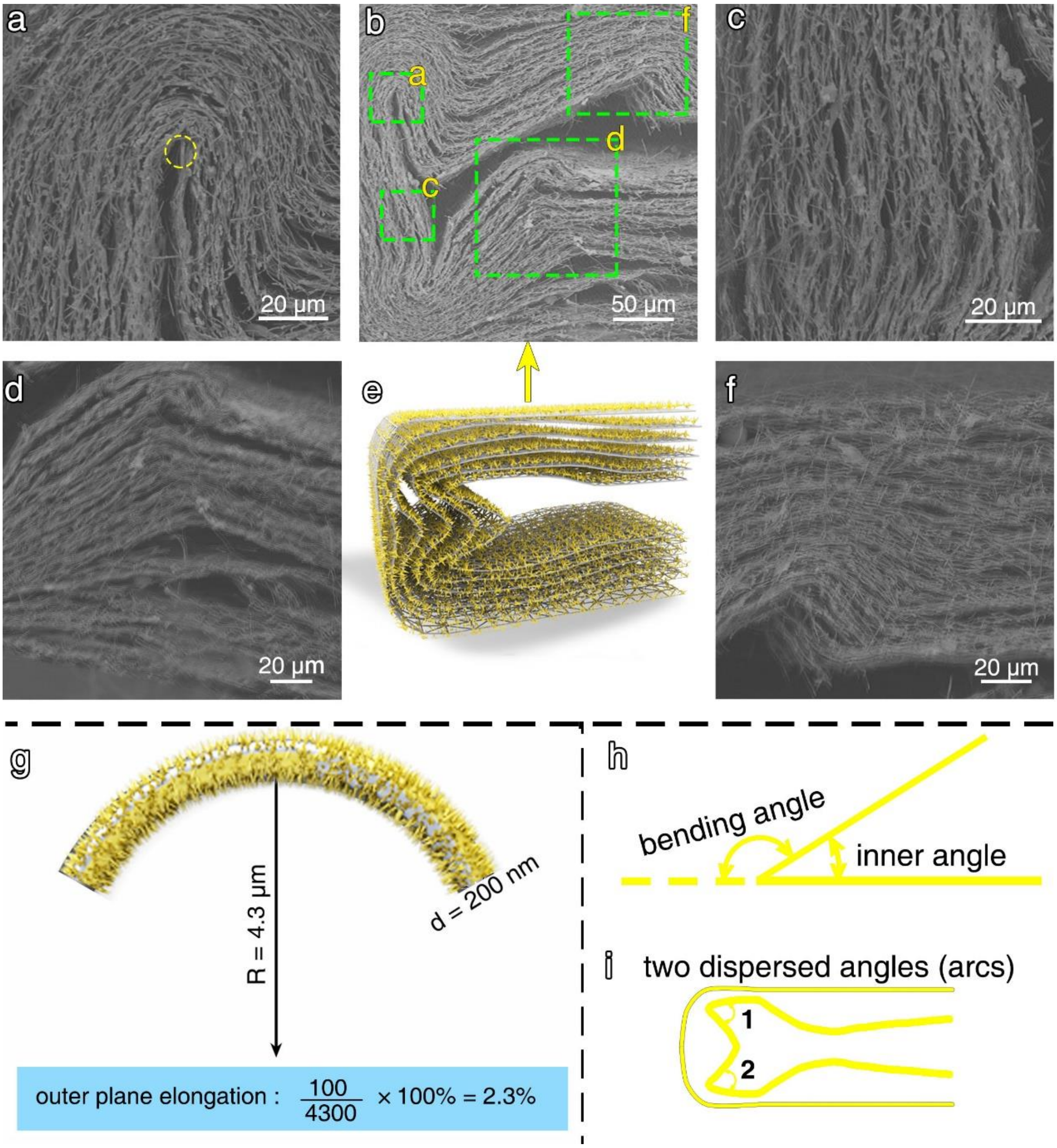

Figure S29. Structure analysis of folded SFCFe. (a) SEM image of formed bending arc. (b) SEM image of completely folded structure. (c, d, f) SEM images of bulged layers in the folded structures (b). (e) Schematic of the folded structures. (g) The largest elongation of a composite nanofiber at the completely folded status of SFCFe. (h) Schematic of formed one bending angle and corresponding inner angle in the folding of unfoldable materials. (i) Schematic of two dispersed arcs formed in SFCFe.

By calculation, the outer plane only has a largest elongation of 2.3% when completely folded. Generally, for unfoldable materials, only one inner acute angle forms during folding, which results in the materials damage until fracture due to too large stress. By contrast, our SFCFe will generate two

dispersed arcs to avoid the formation of inner acute angle to decrease the stress and realize super-folable property.

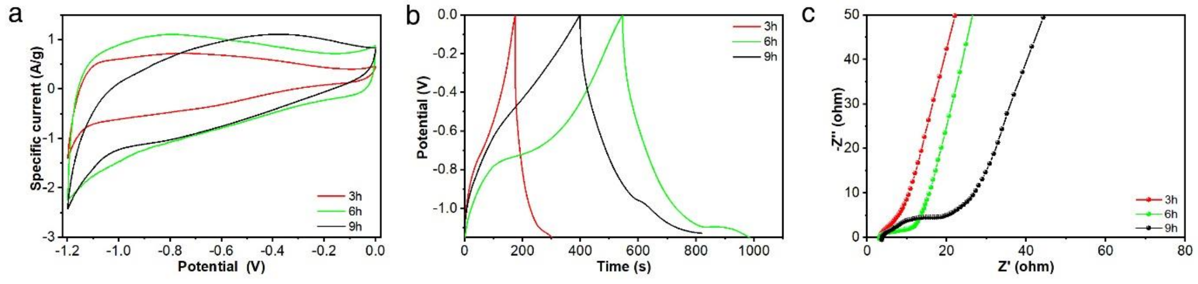

Figure S30. Comparative CV (a), GCD (b), EIS (c) curves of C/FeOOH at different synthesis time.

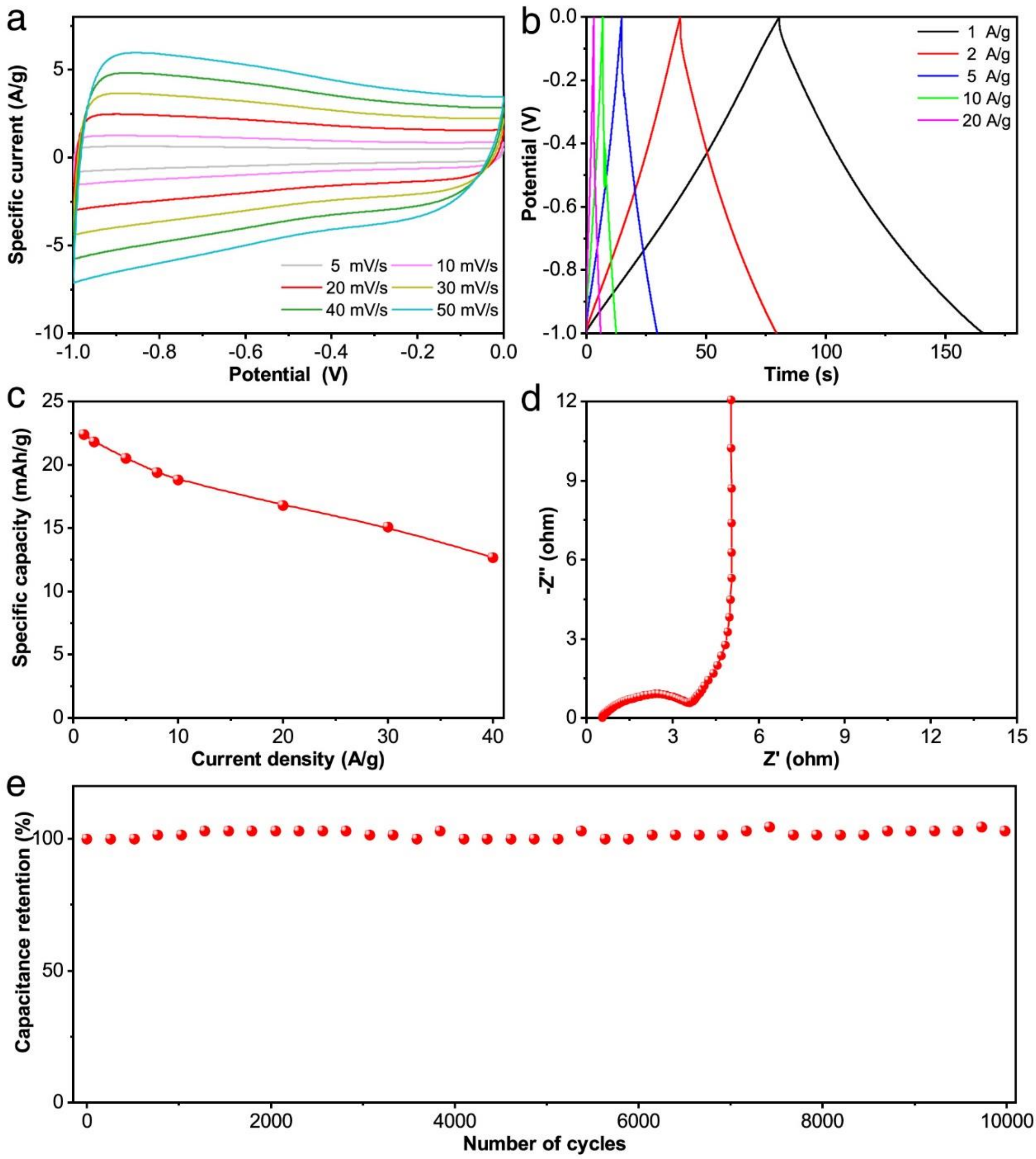

Figure S31. Electrochemical property of C-web substrate in 6M KOH in a three-electrode system. (a) CV curves. (b) GCD curves. (c) Specific capacitance values calculated by GCD curves. (d) EIS curve. (e) Cycling curve.

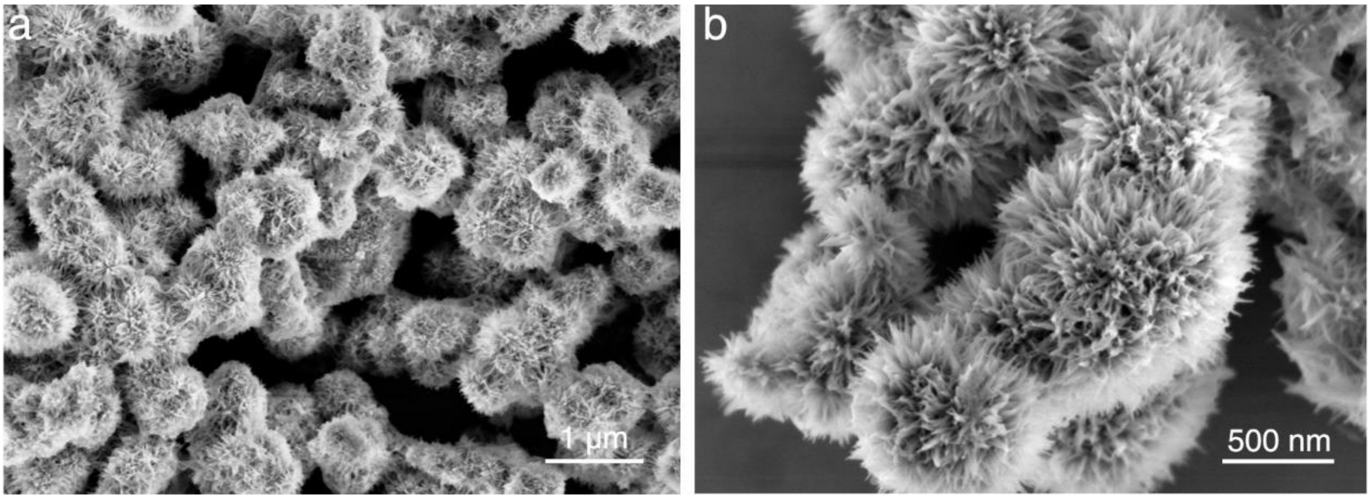

Figure S32. SEM images of pure FeOOH.

Result shows that without the C-web substrate, the FeOOH nanocones will assemble into spheres, which may be not favorable for their full use for electrochemical reactions.

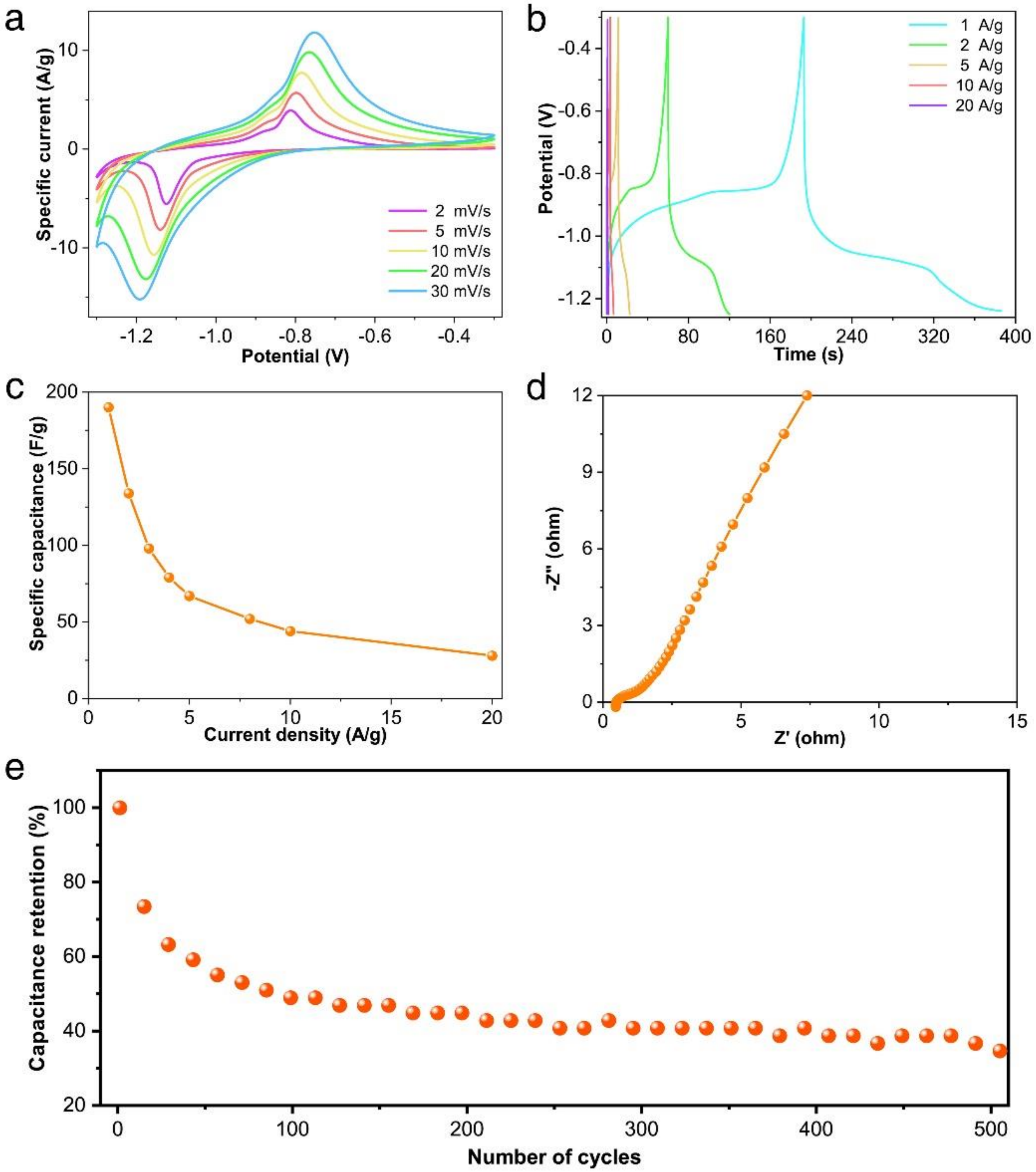

Figure S33. Electrochemical property of pure FeOOH in 6M KOH in a three-electrode system. (a) CV curves. (b) GCD curves. (c) Specific capacitance values calculated by the GCD curves. (d) EIS curve. (e) Cycling curve.

Table S1 Electrochemical property of reported FeOOH flexible electrodes for aqueous energy storage devices

Flexible FeOOH electrode	electrolyte	Specific capacitance / capacity	Rate	Cycling
Rice-like FeOOH/ stainless steel [1]	1M NaOH	444 F/g @ 5 mV/ s	/	/
Amorphous FeOOH/MnO_2/PET [2]	1M Na_2SO_4	350 F/g @ 0.5A/g	160 F/g @ 20A/g	95.6%@10000cycles
Ti-doped FeOOH QD/graphene /bacterial cellulose [3]	1M Na_2SO_4	236 F/g @ 2 mV/ s	/	94.7%@6000cycles
SFCFe (this work)	6M KOH	464 F/g @ 1 A/g (152 mAh/g@1 A/g)	66mAh/g@10A/g (200 F/g @ 10 A/ g)	84%@3000cycles

Video S1.

Strict standard of repeated true-folding test using folding machine. This video shows the strict repeated true-folding process: the two folded parts of the sample are folded to 180° and completely cling.

References:

[1] A. V. Thakur, B. J. Lokhande, *Chem. Pap.* **2018**, *72*, 1407.

[2] Q. Lu, L. Liu, S. Yang, J. Liu, Q. Tian, W. Yao, Q. Xue, M. Li, W. Wu, *J. Power Sources* **2017**, *361*, 31.

[3] R. Liu, L. Ma, G. Niu, X. Li, E. Li, Y. Bai, Y. Liu, G. Yuan, *Part. Part. Syst. Char.* **2017**, *34*.
